# Bridging data gaps of rare conditions in ICU: a multi-disease adaptation approach for clinical prediction

**DOI:** 10.1038/s41746-025-02176-y

**Published:** 2026-01-03

**Authors:** Mingcheng Zhu, Yu Liu, Zhiyao Luo, Tingting Zhu

**Affiliations:** Department of Engineering Science, University of Oxford, Oxford, UK

**Keywords:** Computational biology and bioinformatics, Diseases, Health care, Mathematics and computing, Medical research

## Abstract

Artificial Intelligence has revolutionised critical care for common conditions. Yet, rare conditions in the intensive care unit (ICU), including recognised rare diseases and low-prevalence conditions in the ICU, remain underserved due to data scarcity and intra-condition heterogeneity. To bridge such gaps, we developed KnowRare, a domain adaptation-based deep learning framework for predicting clinical outcomes for rare conditions in the ICU. KnowRare mitigates data scarcity by initially learning condition-agnostic representations from diverse electronic health records through self-supervised pre-training. It addresses intra-condition heterogeneity by selectively adapting knowledge from clinically similar conditions with a developed condition knowledge graph. Evaluated on two ICU datasets across five clinical prediction tasks (90-day mortality, 30-day readmission, ICU mortality, remaining length of stay, and phenotyping), KnowRare consistently outperformed existing state-of-the-art models. Additionally, KnowRare demonstrated superior predictive performance compared to established ICU scoring systems, including APACHE IV and IV-a. Case studies further demonstrated KnowRare’s flexibility in adapting its parameters to accommodate dataset-specific and task-specific characteristics, its generalisation to common conditions under limited data scenarios, and its rationality in selecting source conditions. These findings highlight KnowRare’s potential as a robust and practical solution for supporting clinical decision-making and improving care for rare conditions in the ICU.

## Introduction

Rare conditions in the intensive care unit (ICU), including both formally classified rare diseases and low-prevalence conditions in the ICU^1^, can be life-threatening or chronically debilitating, contributing to a high burden on health systems^2^. Patients with rare conditions often face challenges such as limited access to specialised clinical expertise, frequent misdiagnoses, and prolonged diagnostic delays^3,4^. These factors collectively contribute to worse clinical outcomes, such as a longer stay in the ICU, higher readmission rates, and increased post-discharge mortality compared to common conditions^5,6^. Consequently, rare conditions place a greater per-patient strain on critical care resources than common conditions.

Although artificial intelligence, especially deep learning (DL), has significantly advanced critical care analytics for common conditions such as septic shock and heart failure^7,8^, its application to rare conditions in the ICU remains limited. Existing DL models for common conditions frequently underperform for rare conditions due to insufficient training data, preventing the development of robust and generalisable predictive models^9^. Adding to this problem, the geographic dispersion of patients contributes to the variability in clinical practices and observations across institutions^5^. Meanwhile, rare conditions often exhibit complex clinical manifestations due to multisystem involvement, affecting multiple organs or physiological pathways^2^. Collectively, these factors result in substantial intra-condition heterogeneity.

Recent efforts to improve predictive performance for rare conditions focused mainly on overcoming data scarcity, employing approaches such as few-shot learning^10,11^, federated learning^12,13^, large-scale pre-training^9,14–17^, and synthetic data generation^18,19^. Although these approaches have shown potential in mitigating data scarcity, they often overlook intra-condition heterogeneity. Moreover, existing methods develop one-size-fits-all models, which can work for all conditions or a mixture of conditions. This paradigm further compromises model performance, especially given the diverse presentations and pathophysiology of rare conditions^20^. Therefore, a model designed to bridge the gaps caused by data scarcity and intra-condition heterogeneity in the prediction of outcomes for rare conditions is critical.

In this study, we introduce KnowRare, a DL framework specifically designed to bridge the challenges posed by data scarcity and intra-condition heterogeneity in rare conditions. To address data scarcity, KnowRare first learns condition-agnostic representations through self-supervised pre-training across diverse conditions. To address intra-condition heterogeneity, KnowRare employs knowledge-guided domain adaptation, enabling it to learn robust representations that capture the heterogeneity within the rare conditions. This adaptation is guided by a condition knowledge graph (KG), which encodes clinical similarities among conditions and enables the selective transfer of relevant knowledge from clinically similar conditions. The framework comprises three key modules: condition-agnostic pre-training to establish general time-series representations, knowledge-guided domain selection to identify clinically similar source conditions, and joint adversarial domain adaptation to align patient-level time-series variables and outcome distributions across the selected conditions. These modules enable KnowRare to provide robust condition-specific predictions for rare conditions in the ICU.

We validated KnowRare for five clinical prediction tasks, including 90-day mortality, 30-day readmission, ICU mortality, remaining length of stay (LoS), and phenotyping, using the MIMIC-III and eICU datasets^21,22^. KnowRare consistently outperformed baseline methods in predicting outcomes for rare conditions, demonstrating superior predictive performance and robustness. These findings highlight the effectiveness of KnowRare in overcoming critical gaps caused by data scarcity and intra-condition heterogeneity. By providing accurate, disease-specific predictions, KnowRare has implications for clinical decision-making, improving patient outcomes, and optimising resource allocation tailored to rare conditions in the ICU.

## Results

### Overview of KnowRare

**Fig. 1:**
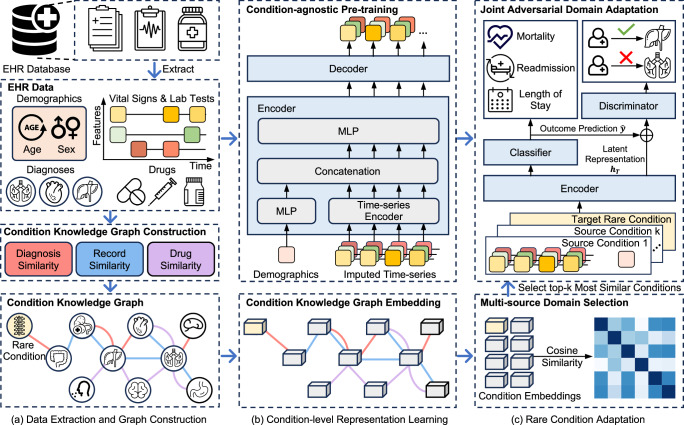
Overview of the KnowRare framework. KnowRare operates in three steps: **a** *Data extraction and graph construction*: Structured EHR data, including demographic data, vital signs, laboratory tests, diagnoses, and drug records, are extracted and preprocessed through aggregation, imputation, and normalisation. Condition similarities are quantified from three perspectives: diagnosis co-occurrence (categorical), record variable distributions (continuous), and shared drug usage (categorical). These similarities are integrated into a heterogeneous condition knowledge graph (KG), capturing comprehensive clinical relationships. **b** *Condition-level representation learning*: This step involves two modules. The condition-agnostic pre-training module trains a time-series encoder via self-supervision to learn general temporal patterns independent of specific conditions, providing robust initial latent representations. Concurrently, the condition KG embedding module uses KG embedding techniques to generate condition embeddings that represent clinical similarities among conditions. **c** *Rare condition adaptation*: This step optimises patient-level representations. First, the knowledge-guided domain selection module identifies the top-*k* source conditions most similar to the target rare condition by calculating cosine similarity between condition embeddings. Subsequently, the joint adversarial domain adaptation module fine-tunes the pre-trained time-series encoder with the target rare condition and the selected top-*k* source conditions. The encoder produces patient-level latent representations (*h*_*T*_), integrating condition-agnostic knowledge with insights derived from similar source conditions. Based on these latent representations, a classifier predicts clinical outcomes ($\widehat{y}$; mortality, readmission, length of stay, etc.). Concurrently, a discriminator network is trained adversarially to distinguish whether the latent representations and predicted outcomes originate from patients with the target rare condition or the selected source conditions. This adversarial process ensures the encoder generates robust representations, which improve predictive performance for heterogeneous rare conditions in the ICU. The figure is created using Microsoft PowerPoint.

KnowRare (Fig. 1) addresses the challenges of scarcity and heterogeneity in predicting the outcomes of rare conditions by integrating both condition-agnostic pre-training and condition-specific adaptation. The framework consists of three stages. First, structured EHR data are extracted and preprocessed. The similarities of the conditions are then calculated and integrated into a heterogeneous condition KG that captures clinical relationships among conditions in general. Second, KnowRare learns condition-level representations using two modules: (i) the condition-agnostic pre-training module trains a time-series encoder through self-supervised next-step prediction, allowing it to capture general patterns independent of specific conditions, and (ii) the condition KG embedding module generates condition embeddings that represent relationships among conditions. Both modules run in parallel. Third, rare condition adaptation optimises patient-level representations through two steps: (i) multi-source domain selection identifies the top-*k* conditions that are clinically similar to the target rare condition using cosine similarity between condition embeddings, and (ii) joint adversarial domain adaptation fine-tunes the pre-trained encoder with patient samples from both the target rare condition and the selected conditions. This process aligns latent representations and prediction outcomes across domains, enabling accurate patient-level outcome prediction by combining generalisable knowledge with transferable insights from clinically similar conditions.

### Data description

We evaluated KnowRare using two publicly available ICU EHR datasets: MIMIC-III^21^ and eICU^22^, both providing granular clinical records suitable for studying rare conditions in the ICU. MIMIC-III is a large, single-centre critical care database comprising health-related data from 46,520 patients admitted to Beth Israel Deaconess Medical Centre between 2001 and 2012. In contrast, eICU is a multi-centre dataset collected from more than 200 hospitals, offering broader variability in patient demographics, phenotypes, and treatment protocols. Following established conventions^23,24^, we classified conditions as *rare* if their prevalence is fewer than one case per 2000 patients within each dataset. The statistics of the datasets are summarised in Table 1, and the variables extracted from each dataset are detailed in Supplementary Table S1. The condition is defined using the first three levels of ICD-9-CM code, with further details provided in Supplementary D.

**Table 1:** Statistics for EHR databases

Statistic	MIMIC-III	eICU
Number of patients	46,520	139,367
Number of hospital Visits	58,976	166,355
Number of ICU stays	61,532	200,859
Number of hospitals	1 (single-centre)	208 (multi-centre)
Number of level 3 ICD-9-CM codes	587	303
Number of Rare Conditions (Percentage)	383 (65.2%)	192 (63.4%)

For MIMIC-III, we evaluated two prediction tasks: (1) 30-day readmission, predicting whether a patient will be readmitted to the hospital within 30 days after discharge, using the final 48 h of hospital admission data; and (2) 90-day mortality, predicting whether a patient will die within 90 days after discharge, also using the final 48 h of hospital admission data. For eICU, we assessed three tasks: (1) ICU mortality, predicting whether a patient will die during their ICU stay, using the first 24 h of ICU admission data; (2) Remaining LoS, predicting the remaining duration of stay in the ICU using the initial 24 h of data, with targets categorised into 10 intervals: less than 1 day, 1–2 days, 2–3 days, 3–4 days, 4–5 days, 5–6 days, 6–7 days, 7–10 days, 10–14 days, and more than 14 days; and (3) classification of phenotypes, predicting the presence of 25 acute care phenotypes using the first 24 h of ICU admission data. All tasks adhere to established benchmark definitions^25,26^.

We selected the ten least prevalent conditions in the ICU, including both recognised rare diseases and low-prevalence conditions, based on two criteria: (1) prevalence of the condition less than one in 2000 patients^23,27^, and (2) at least one positive outcome sample (e.g., mortality or readmission case) per prediction task evaluated. The conditions were ranked according to their total number of cases, and the least prevalent ones satisfying both criteria were selected until we obtained ten qualifying conditions (the statistics are summarised in Supplementary Tables S2 and S3). To prevent data leakage, patients were split into training (67%), validation (16%), and test (17%) sets, maintaining patient-level separation across sets^9,28^. Additional details on preprocessing steps are provided in Supplementary B. Sensitivity analyses of preprocessing parameters are detailed in Supplementary E and Supplementary F. Model performance for these rare conditions was evaluated using the Area Under the Receiver Operating Characteristic Curve (AUROC) and the Area Under the Precision-Recall Curve (AUPRC), with the latter preferred for class imbalance.

### Performance evaluation of KnowRare

We evaluated the performance of KnowRare across five clinical prediction tasks on two publicly available datasets, comparing it with widely-used methods for ICU outcome prediction and specific methods for limited-data scenarios. KnowRare achieves the highest AUPRC on all five tasks, outperforming both baseline categories (Table 2). In particular, it surpasses the best domain adaptation method (Stable-CRP) by 12.4% in ICU mortality (AUPRC: 0.709 vs. 0.631) and outperforms MetaPred by 17.0% in Remaining LoS (AUPRC: 0.206 vs. 0.176) on the eICU dataset. KnowRare consistently ranks first or second, demonstrating balanced precision-recall trade-offs.

**Table 2:** Performance comparison of KnowRare with other baselines^c^

Method	MIMIC-III				eICU					
	90 Days Mortality		30 Days Readmission		ICU Mortality		Remaining LoS		Phenotyping	
	AUPRC	AUROC	AUPRC	AUROC	AUPRC	AUROC	AUPRC	AUROC	AUPRC	AUROC
LSTM^57^ (single^a^)	0.339 (0.039)	0.432 (0.072)	0.370 (0.044)	0.488 (0.062)	0.427 (0.113)	0.491 (0.106)	0.125 (0.022)	0.459 (0.061)	0.131 (0.009)	0.510 (0.089)
LSTM^57^ (all^b^)	0.725 (0.028)	0.789 (0.022)	0.693 (0.095)	0.744 (0.048)	0.569 (0.039)	0.725 (0.051)	0.124 (0.058)	0.519 (0.037)	0.137 (0.032)	0.555 (0.040)
Transformer^61^ (single)	0.539 (0.130)	0.610 (0.126)	0.439 (0.085)	0.578 (0.073)	0.381 (0.058)	0.521 (0.055)	0.135 (0.017)	0.491 (0.056)	0.152 (0.058)	0.476 (0.074)
Transformer^61^ (all)	0.665 (0.045)	0.751 (0.048)	0.706 (0.031)	0.747 (0.014)	0.636 (0.013)	**0.770** (0.023)	0.090 (0.066)	0.505 (0.009)	0.121 (0.033)	0.597 (0.056)
RETAIN^62^ (single)	0.365 (0.027)	0.493 (0.056)	0.311 (0.019)	0.448 (0.055)	0.415 (0.074)	0.518 (0.042)	0.141 (0.027)	0.514 (0.053)	0.172 (0.022)	0.545 (0.032)
RETAIN^62^ (all)	0.679 (0.058)	0.770 (0.032)	0.545 (0.082)	0.625 (0.086)	0.532 (0.046)	0.733 (0.025)	NA	NA	0.061 (0.041)	0.488 (0.032)
Metapred^10^	0.481 (0.028)	0.618 (0.031)	0.394 (0.044)	0.534 (0.016)	0.557 (0.038)	0.643 (0.024)	0.176 (0.097)	**0.525** (0.025)	0.237 (0.062)	**0.656** (0.026)
RareMed^9^	0.565 (0.028)	0.773 (0.023)	0.686 (0.050)	0.741 (0.052)	0.545 (0.018)	0.625 (0.032)	0.096 (0.039)	0.494 (0.008)	0.168 (0.017)	0.591 (0.039)
SMART^15^	0.610 (0.062)	0.733 (0.035)	0.705 (0.056)	0.721 (0.039)	0.544 (0.027)	0.711 (0.025)	0.059 (0.021)	0.496 (0.019)	0.220 (0.035)	0.565 (0.044)
FADA^63^	0.737 (0.024)	0.784 (0.018)	0.701 (0.044)	0.730 (0.050)	0.521 (0.036)	0.684 (0.030)	0.057 (0.001)	0.500 (0.0)	0.156 (0.032)	0.540 (0.027)
Advdiag^59^	0.717 (0.023)	0.779 (0.004)	0.711 (0.075)	0.730 (0.045)	0.497 (0.026)	0.631 (0.033)	0.094 (0.044)	0.494 (0.030)	0.157 (0.024)	0.532 (0.027)
Stable-CRP^60^	0.726 (0.041)	**0.819** (0.027)	0.646 (0.069)	0.686 (0.062)	0.631 (0.053)	0.691 (0.027)	0.077(0.027)	0.501 (0.026)	0.158 (0.034)	0.546 (0.029)
MANYDG^64^	0.622 (0.054)	0.663 (0.079)	0.637 (0.068)	0.643 (0.038)	0.470 (0.054)	0.632 (0.036)	0.138 (0.009)	0.453 (0.014)	0.210 (0.056)	0.570 (0.010)
*KnowRare* (Ours)	**0.744** (0.051)	0.797 (0.032)	**0.716**(0.031)	**0.749** (0.034)	**0.709**(0.022)	0.757 (0.012)	**0.206** (0.024)	0.509 (0.030)	**0.244** (0.039)	0.609 (0.044)

^a^ (single) refers to training the model only on data of rare conditions.

^b^ (all) refers to training the model on data of all conditions.

^c^ The first method group consists of the baelines for ICU outcome predicitons, while the second group includes domain adaptation baselines. Metrics are reported as mean (std) over five runs with different random seeds. The best results are bolded, and the second best are underlined.

### Performance comparison with ICU scoring systems

To further evaluate the clinical relevance of KnowRare, we compared its performance in the prediction of ICU mortality with established ICU scoring systems, specifically APACHE IV^29^ and APACHE IV-a^30^. These systems are considered gold standards for the prediction of mortality in ICU settings^31^. The analysis focused on the prediction of ICU mortality in the eICU dataset.

For the ICU mortality prediction, KnowRare demonstrated superior performance compared to APACHE IV and APACHE IV-a^29^ (Table 3). KnowRare achieved an AUPRC of 0.709 and an AUROC of 0.757, outperforming APACHE IV (AUPRC: 0.639, AUROC: 0.701) and APACHE IV-a (AUPRC: 0.627, AUROC: 0.695). These results indicate that KnowRare provides improved predictive accuracy for ICU mortality, reinforcing its potential clinical utility over traditional scoring systems.

**Table 3:** Performance comparison of KnowRare against ICU scoring systems^a^

Method	ICU Mortality (eICU)	
	AUPRC	AUROC
APACHE IV^29^	0.639	0.701
APACHE IV-a^30^	0.627	0.695
KnowRare (Ours)	**0.709** (0.022)	**0.757** (0.012)

^a^ Metrics are reported as mean (std) over five runs with different random seeds. The best results are bolded.

### Ablation study

To assess the contributions of different modules in KnowRare, we conducted an ablation study by independently removing three key modules: (1) condition-agnostic pre-training, (2) knowledge-guided domain selection, and (3) joint adversarial domain adaptation. The statistical significance of performance differences was evaluated using two-tailed t-tests^32^. The results, summarised in Table 4, indicate that the removal of any module consistently leads to decreased AUROC or AUPRC across all tasks, demonstrating the necessity of each module. Among these, the knowledge-guided domain selection module proved to be the most critical, as its removal resulted in consistent reductions in performance across all tasks on both datasets, with notable performance drops observed in the multi-centre eICU dataset (the mean of AUPRC reduced from 0.709 to 0.573 for ICU mortality and from 0.206 to 0.065 for remaining LoS). Removing condition-agnostic pre-training also consistently reduced performance across most tasks, decreasing the mean of AUPRC for the ICU mortality prediction in MIMIC-III from 0.744 to 0.640. Similarly, removing joint adversarial domain adaptation markedly decreased the mean of AUPRC in the 30-day readmission task in MIMIC-III from 0.716 to 0.481.

**Table 4:** Ablation Study of KnowRare^a^

Method	MIMIC-III				eICU					
	90 Days Mortality		30 Days Readmission		ICU Mortality		Remaining LoS		Phenotyping	
	AUPRC	AUROC	AUPRC	AUROC	AUPRC	AUROC	AUPRC	AUROC	AUPRC	AUROC
w/o Pre-training	0.640 (0.090)	0.730 (0.054)	0.690 (0.067)	0.747 (0.031)	0.628^*^ (0.049)	0.693^***^ (0.009)	0.134^**^ (0.011)	0.483 (0.023)	0.237 (0.057)	0.629 (0.032)
w/o Domain Selection	0.660^*^ (0.043)	0.793 (0.015)	0.699 (0.081)	0.776 (0.068)	0.573^***^ (0.026)	0.661^**^ (0.037)	0.065^***^ (0.017)	0.495 (0.011)	0.146^**^ (0.016)	0.575 (0.047)
w/o Domain Adaptation	0.679 (0.061)	0.785 (0.589)	0.481^*^ (0.139)	0.598^*^ (0.089)	0.713 (0.025)	0.725^**^ (0.013)	0.151^**^ (0.018)	0.512 (0.016)	0.253 (0.049)	0.586 (0.044)
*KnowRare*	0.744 (0.051)	0.797 (0.032)	0.716 (0.031)	0.749 (0.034)	0.709 (0.022)	0.757 (0.012)	0.206 (0.024)	0.509 (0.030)	0.244 (0.039)	0.609 (0.044)

^a^ Metrics are reported as mean (std) over five runs with different random seeds. *denotes statistical significance compared with KnowRare using the t-test over five runs with different random seeds. * for *p* < 0.05, ** for *p* < 0.01, and *** for p < 0.001.

### Case studies: evaluation of KnowRare’s adaptability and generalisation

This section presents three case studies designed to evaluate KnowRare’s adaptability and generalisation in real-world clinical settings. Specifically, we conducted experiments to assess: (1) the number of source conditions required by KnowRare to adapt effectively across different datasets and prediction tasks; (2) the impact of condition KG sparsity on KnowRare’s adaptability to different dataset characteristics; and (3) KnowRare’s ability to generalise robustly to common conditions under limited training data scenarios. We also evaluate the discriminative performance and calibration of KnowRare (Supplementary G and H).

**Fig. 2:**
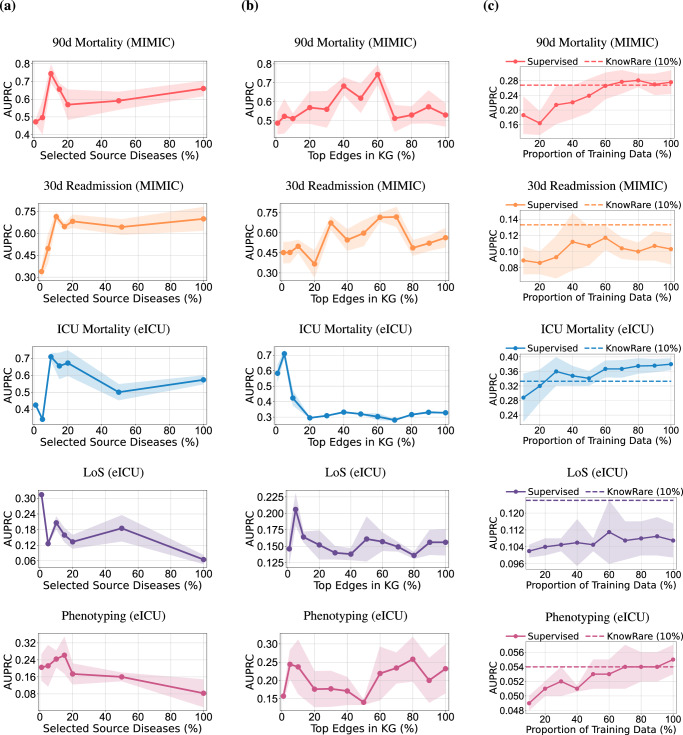
Case studies: Evaluating KnowRare’s adaptability and generalisation in clinical practice. Studies include: **a** Analysis of required source conditions: Assessing KnowRare’s adaptability to different hospital datasets and clinical prediction tasks by varying the proportion of source conditions included from 1% to 100%. **b** Impact of condition KG sparsity: Assessing KnowRare’s sensitivity and adaptability to varying KG completeness by retaining different proportions of top-weighted edges (from 1% to 100%) in the KG. **c** Generalisation to common conditions under limited-data scenarios: Evaluating KnowRare’s robustness by training it with only 10% of the available septicemia data, while the LSTM baseline model uses septicemia data ranging from 10% to 100%. Rows correspond to the five prediction tasks: (1) 90-day mortality prediction after hospital discharge (MIMIC-III), (2) 30-day readmission prediction after hospital discharge (MIMIC-III), (3) ICU mortality prediction (eICU), (4) Remaining length of stay prediction (eICU), and (5) Phenotyping prediction (eICU). Points represent mean values, and shaded regions indicate standard deviation over five runs. Plots are generated using matplotlib (Python).

To evaluate the influence of source condition diversity on domain adaptation, we varied the proportion of source conditions selected for KnowRare training, ranging from 1% to 100% of the available source conditions. The objective was to determine whether the broader inclusion of source conditions enhances generalisation or introduces confounding. The results (Fig. 2(a)) reveal a non-linear relationship between the quantity of the source condition and the performance of the model. Initially, increasing the proportion of source conditions improves the accuracy of the task, with peak performance occurring at 10-20% across tasks. Beyond this threshold, performance declines sharply for ICU mortality, LoS, and phenotyping predictions in the multi-centre setting, eventually stabilising at higher proportions. In particular, for LoS prediction, minimal source diversity (1% of conditions) yields superior results compared to larger selections.

In addition, we investigated the effect of the completeness of the condition KG. This was achieved by iteratively retaining only the top n% of the weighted edges (ranging from 1% to 100%) in the KG and re-evaluating the KnowRare framework. The experiment aimed to identify whether sparser, high-confidence relationships or denser, inclusive graphs better support adaptation in clinical prediction tasks. The optimal proportion of retained edges differs markedly between datasets (Fig. 2(b)). For the multi-centre eICU cohort, performance peaks when the top 5% of KG edges are included, except for the phenotyping prediction task. The single-centre MIMIC-III dataset achieves maximal AUPRC with 60-70% of edges. Beyond these thresholds, inclusion of lower-weighted edges correlates with progressive performance degradation. One outlier appears at the phenotyping prediction task, which exhibits a dual-peak pattern in the eICU dataset, achieving optimal performance at both 5% and 80% edge retention.

We further evaluated KnowRare’s applicability to common conditions in scenarios characterised by limited training data. Common conditions can also experience data scarcity due to practical constraints. To examine whether KnowRare could generalise to such situations, we experimented with septicaemia, a common ICU condition, deliberately restricting the training set to only 10% of the available samples. This setting assumes access to only a small amount of data that is insufficient for condition-agnostic pre-training. Therefore, the pre-training module was disabled, ensuring an accurate evaluation of KnowRare’s ability to exploit general clinical knowledge under severe resource constraints. Our results (Fig. 2(c)) demonstrated that KnowRare achieved comparable or superior performance to a standard LSTM model trained on all available data on 90-day mortality, 30-day readmission, and remaining LoS. In ICU mortality and phenotyping prediction tasks, the standard LSTM model only surpassed KnowRare’s performance when trained with at least three times more labelled data. These findings underscored KnowRare’s potential to effectively leverage clinical insights from similar conditions, thereby improving predictive performance in data-limited clinical scenarios.

### Explanability analysis

**Fig. 3:**
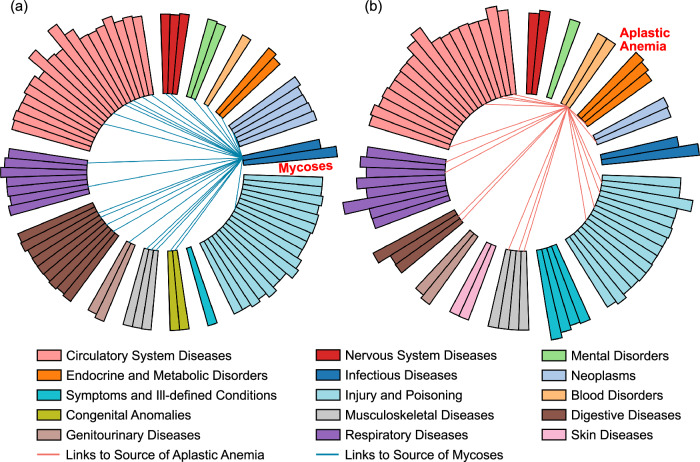
Visualisation of conditions selected by KnowRare as source conditions for predicting mycoses in MIMIC-III and aplastic anaemia (AA) in eICU. **a** Mycoses; **b** AA. Conditions are categorised according to the ICD-9-CM classification system, with each category represented by a distinct colour. The height of each bar corresponds to the number of patients diagnosed with each condition, indicating their relative prevalence within the datasets. This visualisation illustrates how KnowRare selects clinically similar source conditions different from ICD-9-CM hierarchical relationships. The visualisation is generated using matplotlib (Python).

To evaluate the explanability of KnowRare’s selection of source conditions, we visualised its choices for two rare conditions: mycoses, a low-prevalence condition in MIMIC-III, and aplastic anaemia (AA), a rare recognised condition in eICU. For each case, we included the 40 most prevalent conditions, the 10 least prevalent conditions, and the subset of conditions selected by KnowRare (Fig. 3). This analysis aimed to determine whether the framework prioritises hierarchy from the ICD-9-CM coding system or instead identifies clinical similarities directly from the heterogeneous EHR data. A summary of all rare conditions provides a quantitative overview of the selected source conditions (Supplementary Table S5).

The analysis yielded a key observation. 80.8% of the source conditions in MIMIC-III and 90.5% in eICU selected by KnowRare belonged to ICD-9-CM categories different from those of the target rare condition (Table S8). Specifically, for mycoses, KnowRare predominantly selected source conditions from circulatory system disorders (ICD-9-CM 390-459), even though fungal infections are classified under infectious conditions (ICD-9-CM 001-139). Likewise, for AA, the model primarily selected source conditions from injury and poisoning (ICD-9-CM 800-999), despite AA being categorised as a blood disorder (ICD-9-CM 280-289). These results indicated that KnowRare captured clinical similarities directly from heterogeneous patient data rather than relying solely on established ICD-9-CM hierarchies.

## Discussion

In this study, we proposed KnowRare, a DL framework designed to improve the prediction of clinical outcomes for rare conditions in the ICU. KnowRare addresses two critical challenges, data scarcity and intra-condition heterogeneity, by combining condition-agnostic pre-training with knowledge-guided domain adaptation. Evaluation of two publicly available ICU datasets (MIMIC-III and eICU) demonstrated that KnowRare consistently outperformed baseline methods, achieving improvements of up to 17.0% in AUPRC. The consistent improvement across multiple clinical tasks, including 90-day mortality after discharge, 30-day readmission after discharge, ICU mortality, ICU LoS, and phenotyping, demonstrates KnowRare’s potential to improve clinical decision-making for rare conditions in ICUs.

We observed a clear “data-volume paradox" with standard methods in our experiments (Table 2): training exclusively on target rare conditions resulted in significantly inferior performance, whereas aggregating data from multiple conditions improved results in single-centre scenarios but degraded performance in multi-centre scenarios. This paradox underscores the necessity for careful selection of aggregated data sources to ensure that increased data volume does not compromise the quality and clinical relevance of training data. KnowRare addresses this issue through knowledge-guided domain adaptation, selectively identifying and utilising only the most similar source conditions. This targeted approach enhances data volume without amplifying irrelevant noise, contributing significantly to KnowRare’s effectiveness.

Specialised methods for rare conditions demonstrated strong performance in individual prediction tasks, but lacked robustness when evaluated across multiple clinical outcomes (Table 2). In contrast, KnowRare showed consistent effectiveness across various clinical tasks. This improved generalisation was primarily driven by its two-stage training strategy: initially capturing generalisable temporal patterns through condition-agnostic pre-training, followed by fine-tuning with knowledge-guided domain adaptation to selectively activate condition-specific knowledge for each rare condition. This finding is consistent with related research for cases with limited data^9,10^. Specifically, the condition-agnostic pre-training process enables KnowRare to establish robust baseline representations capable of generalising effectively to data-scarce conditions. Furthermore, the knowledge-guided domain adaptation, particularly through the domain selection module, substantially addresses intra-condition heterogeneity (Table 4). By selecting clinically similar conditions based on the condition KG, this module exposes KnowRare to diverse clinical patterns without introducing excessive noise. Collectively, KnowRare provides a step toward addressing the challenges of data scarcity and intra-condition heterogeneity of rare conditions in the ICU.

For the most critical ICU mortality prediction, KnowRare outperformed traditional ICU scoring systems, including APACHE IV and IV-a. These systems typically rely on large cohorts that primarily comprise common conditions, which limits their effectiveness for rare conditions. In contrast, KnowRare’s tailored selection of clinically similar conditions and knowledge-guided adaptation processes enabled superior performance specifically for rare conditions.

The ablation study shows that the contributions of the KnowRare modules vary across prediction tasks and datasets. Although some modules fail to contribute consistently across tasks, none of their removals led to a significant performance decline, highlighting the synergistic value of combining modules for different scenarios. However, task-specific patterns reveal insightful differences. 90-day mortality prediction benefits only from domain selection, consistent with the fact that long-term mortality is linked to similar comorbidities across clinically similar conditions^33^. 30-day readmission benefits mainly from domain adaptation, as aligned features across conditions capture discharge processes and early post-discharge care, which are strongly linked to readmission risk^34^. ICU mortality prediction benefits from all modules, as short-term outcomes in the ICU are dominated by acute pathophysiology and rapidly evolving critical events at the beginning of ICU stay^35^. In this case, pre-training captures shared physiological patterns, domain selection identifies clinically similar acute conditions, and domain adaptation helps identify evolving critical events. Remaining LoS in eICU improves with all modules, with the most significant contribution from domain selection. This is potentially because non-clinical operational factors vary widely across hospitals, and domain selection helps reduce this variance^36,37^. Phenotyping task benefits only from domain selection, as leveraging clinically similar conditions helps the model better define phenotype boundaries^38^. Pre-training and domain adaptation can negatively affect phenotyping. Pre-training on the full condition cohort may introduce inter-institutional heterogeneity^39^, while domain adaptation may blur critical distinctions between phenotypes by forcing alignment of latent features^40^.

The selection of source conditions by KnowRare was primarily based on data-driven relationships rather than strictly following the ICD coding hierarchies (Fig. 3). This approach is particularly effective for rare conditions, where standard ICD categories often do not capture nuanced cohort-specific similarities. By uncovering clinically meaningful relationships, KnowRare not only improves interpretability but also provides clinicians with insights into how knowledge is transferred across conditions. Specifically, for mycoses in MIMIC-III, many selected source conditions were related to circulatory system diseases (ICD-9-CM 390-459), which is clinically plausible given that fungal infections can spread through the bloodstream after initial invasion^41,42^. Similarly, for AA in eICU, KnowRare primarily selected source conditions related to injury and poisoning (ICD-9-CM 800-999), consistent with the fact that AA can arise from prolonged exposure to toxic agents such as chemotherapy or radiotherapy, which progressively damage haematopoietic stem cells^43^.

Interestingly, our finding contradicts the conventional idea that using more data leads to better performance until saturation^44,45^. Instead, optimal performance for KnowRare was achieved by selecting the top 10% most similar conditions for domain adaptation. Including more data for training introduced excessive noise, hindering effective domain adaptation. Particularly in the multi-centre eICU dataset, significant performance degradation occurred beyond 50% data inclusion (Fig. 2(a)). However, an exception was the LoS prediction task, where restricting the data to only 1% of the most similar conditions optimally prevented the overfitting of irrelevant clinical patterns. Unlike mortality or phenotyping, which are driven by underlying pathophysiology and acute clinical events, LoS is strongly affected by non-clinical operational factors such as discharge policies, bed availability, and resource allocation^36,37^. These factors introduce substantial inter-centre variability, making the inclusion of additional source conditions more likely to degrade model performance.

Our findings also show KnowRare’s capability to adapt to diverse ICU scenarios by adjusting the retention threshold of relationships in the condition KG (Fig. 2(b)). Optimal performance in multi-centre datasets (eICU) was achieved by retaining the strongest 5% of the graph edges, while single-centre datasets (MIMIC-III) benefited from the retention of a higher proportion (60–70%). An exception emerged in phenotyping prediction, which exhibited a dual-peak performance pattern. Phenotyping prediction benefited from both highly confident, tightly coupled clinical relationships at lower retention thresholds and broader, weaker connections at higher thresholds. This unique pattern can be caused by the distinct nature of phenotyping prediction, where multiple concurrent patterns must be identified simultaneously^26^. Besides the dataset-specific threshold, subtle, long-range clinical relationships can enhance the identification of meaningful phenotypes overlooked by strong connections, therefore contributing to better performance in phenotyping prediction.

Beyond rare conditions, KnowRare extended its utility to common conditions that experienced data scarcity. Our evaluation with limited training samples for septicaemia demonstrated that KnowRare could achieve comparable or superior predictive performance compared to standard DL models trained on all data for the common condition. This result highlights KnowRare’s potential utility in clinical settings facing real-world data constraints due to operational or ethical limitations^46,47^.

KnowRare has the potential to be integrated into ICU workflows to stratify risk and support decision-making for rare conditions, similar to established scoring systems such as APACHE IV^30^. Specifically, early and accurate risk stratification in the ICU can help clinicians identify high-risk patients with rare conditions, where records are often poorly characterised^3,4^. Hence, time-sensitive interventions may have an even greater impact on survival compared to common conditions^48^. Second, reliable predictions of LoS and readmission risk are especially valuable to allow a more efficient allocation of scarce ICU beds and staff for rare conditions, where patients are more likely to have prolonged stays in the ICU or a higher readmission risk^5^. Third, improved phenotyping based on the first 24 h of admissions to the ICU could help clinicians identify subgroups of patients who may benefit from personalised management strategies, especially for patients with rare conditions^49^.

Several limitations of our study should be acknowledged. Firstly, our reliance on ICD-9 coding constrained the granularity of condition categorisation, although this limitation arose directly from the datasets used (MIMIC-III and eICU). This limited granularity could reduce the accuracy of capturing subtle clinical distinctions among rare conditions, potentially limiting the effectiveness of knowledge-guided domain adaptation. Secondly, differences in ICD-CM recording methods restricted our ability to evaluate identical tasks across datasets. In MIMIC-III, ICD-CM codes are only available at discharge, providing a retrospective summary of the entire stay in the ICU rather than reflecting the evolving clinical state during admission. This constrains our analyses to post-discharge clinical predictions and may reduce the generalisability of findings to settings where real-time diagnostic processes are critical. In contrast, eICU records time-resolved ICU diagnosis codes, which better represent the ongoing diagnostic process and thus support more generalisable evaluation.

In conclusion, KnowRare effectively bridges data gaps to predict the outcomes of rare conditions in the ICU by integrating general knowledge and selectively adapting insights from clinically similar conditions. Validated through extensive experiments on real-world ICU datasets, KnowRare demonstrates robust predictive performance and strong potential to support clinical decision-making. Future research should focus on prospective validation in clinical settings and investigate opportunities for integration within broader healthcare systems to further enhance care for rare ICU conditions.

## Methods

### Data processing

To ensure a comprehensive representation of patient data across the datasets, we extracted variables, including demographic data, vital signs, and laboratory tests, from MIMIC-III and eICU (Supplementary Table S1). The extraction process involves multiple steps, including cohort selection, variable aggregation, missing value imputation, and normalisation. First, we exclude patients with a hospital stay of less than 48 h in the MIMIC-III dataset and those with an ICU stay of less than 24 h in the eICU dataset to ensure sufficient data availability for inference. Then, each patient is assigned a primary diagnosis based on the ICD-9-CM coding system, using the first three levels of their ICD-9-CM code. To maintain a sufficient sample size for a robust evaluation, we exclude conditions with fewer than ten patients. After applying these criteria, the final dataset consists of 38,360 MIMIC-III samples and 72,536 eICU samples. We then split each dataset into training sets (67%), validation sets (16%), and test sets (17%) at the patient level, ensuring that all ICU stays belonging to the same patient were assigned to the same subset. In addition, we stratify the patients for each condition across splits to ensure a representative distribution in the training, validation, and test sets.

After selecting the relevant cohorts of patients, we extract demographic variables, including age, gender, and race, from patient metadata. Vital signs and laboratory test measurements are obtained from structured EHR tables. For MIMIC-III, we use the recorded values from the last 48 h of hospital admission, while for eICU, we extract recorded values from the first 24 h of ICU admission. The difference in these time windows reflects the distinct prediction tasks of each dataset. In MIMIC-III, we focus on the outcomes of post-ICU discharge. The last 48 h provide an optimal balance between information content and computational efficiency, and were widely used as the default time window for post-discharge prediction^25,50,51^. In the eICU, we focus on outcomes in the ICU, where the first 24 h capture the patient’s acute physiological response to critical illness and support early clinical decision-making^52^.

To create a structured time-series representation, we aggregated the extracted variables into fixed time resolutions following established benchmarks^25,26^. For MIMIC-III, we segment the time-series data into 2-hour windows and compute the mean value within each window, resulting in 24 time steps spanning the last 48 h of hospital admission. For eICU, we apply a similar method using 1 h windows, also producing 24 time steps covering the first 24 h of ICU admission. If no recorded values are available within a given time step, they are left as missing values at this stage.

To handle missing values, we adopt the last observation carried forward / next observation carried backward (LOCF/NOCB) strategy^53,54^. First, we apply forward imputation, where missing values are replaced with the last available measurement for the same patient. If no previous value exists, we apply backwards imputation, filling in missing values using the next available measurement from the same patient. After these steps, any remaining missing values are imputed using the mean value of the corresponding variables computed from the training set. This ensures that the validation and test sets remain independent and do not incorporate statistical information from unseen data.

Finally, to standardise the variable scales, we normalise all continuous variables using Z-score normalisation^55^, where each variable is transformed using the mean and standard deviation computed from the training set. This normalisation step ensures consistent variable distributions across different datasets and tasks while preventing data leakage.

### Baseline models

To comprehensively evaluate KnowRare, we compared its performance against two categories of baseline methods: standard models commonly used for clinical prediction tasks and specialised methods explicitly designed for data-scarce scenarios. Detailed descriptions of the baseline methods are summarised in Table 5.

**Table 5:** Baseline methods included in this study

Name	Description
LSTM^57^	Long short-term memory network for modelling EHR temporal dependencies.
Transformer^61^	Self-attention architecture capturing long-range variable interactions.
RETAIN^62^	Interpretable attention-based recurrent neural network for clinical time-series.
MetaPred^10^	Gradient-based meta-learning framework for few-shot condition diagnosis.
RareMed^9^	Pre-training on clinical notes to enhance rare condition medication recommendations.
SMART^15^	Self-supervised learning via masked reconstruction of EHR data.
FADA^63^	Few-shot classification via adversarial domain alignment.
AdvDiag^59^	Adversarial training to handle cross-population diagnostic distribution shifts.
Stable-CRP^60^	Patient data reweighting for stable predictions amidst temporal shifts.
MANYDG^64^	Learning domain-invariant representations to generalise across patient domains.

### Condition knowledge graph construction

To identify clinically similar source conditions, a heterogeneous condition KG ${\mathcal{G}}=({\mathcal{V}},{\mathcal{E}})$ is constructed from the EHR database, where ${\mathcal{V}}$ represents the set of conditions, ${\mathcal{E}}$ defines the types of relationships that capture similarities of the condition. The graph encodes three types of relationships to model condition similarities.

#### Diagnosis similarity

The diagnosis-based relation captures co-occurrence patterns between conditions within diagnosis records. In EHRs, multiple ICD-CM codes are assigned during a patient visit, resulting in frequent co-occurrence of conditions within the same diagnosis record. A triplet $({v}_{i},{r}_{1},{v}_{j})\in {\mathcal{E}}$ is established between conditions ${v}_{i},{v}_{j}\in {\mathcal{V}}$ if they frequently appear together in patient records. The edge weight is computed as the normalised co-occurrence frequency:

1 $${w}_{ij}^{({r}_{1})}=\frac{\,{\rm{CoOcc}}({v}_{i},{v}_{j})}{{\sum }_{k}{\rm{CoOcc}}\,({v}_{i},{v}_{k})},$$

where CoOcc(*v*_*i*_, *v*_*j*_) represents the number of times conditions *v*_*i*_ and *v*_*j*_ appear together in the same diagnosis record.

#### Record similarity

The record-based relation models condition similarity based on statistical patterns in patient variables. Each condition $v\in {\mathcal{V}}$ is represented by a vector **s**_*v*_, calculated as the mean and standard deviation:

2 $${{\bf{s}}}_{v}=\left[\,{\rm{mean}}\,({{\mathcal{X}}}_{v}),\,{\rm{std}}\,({{\mathcal{X}}}_{v})\right],$$

where ${{\mathcal{X}}}_{v}=\{{{\bf{X}}}_{p}| p\in {{\mathcal{P}}}_{v}\}$ denotes the set of time-series variables of all patients ${{\mathcal{P}}}_{v}$ diagnosed with condition *v*, and *p* refers to an individual patient with corresponding variables **X**_*p*_. The mean and standard deviation are computed element-wise across all patient records.

The similarity weight between conditions *v*_*i*_ and *v*_*j*_ is computed using the inverse L2 distance:

3 $${w}_{ij}^{({r}_{2})}=\frac{1}{1+{||{{\bf{s}}}_{{v}_{i}}-{{\bf{s}}}_{{v}_{j}}||}_{2}}.$$

To retain meaningful relationships, only the top 50% of the highest-weighted record-based connections are preserved.

#### Drug similarity

The drug-based relationship captures the similarity of the conditions based on the use of shared medications. For each condition $v\in {\mathcal{V}}$, the weight of the relationship between conditions *v*_*i*_ and *v*_*j*_ is calculated using the Jaccard similarity of their drug sets:

4 $${w}_{ij}^{({r}_{3})}=\frac{| {{\mathcal{D}}}_{{v}_{i}}\cap {{\mathcal{D}}}_{{v}_{j}}| }{| {{\mathcal{D}}}_{{v}_{i}}\cup {{\mathcal{D}}}_{{v}_{j}}| },$$

where ${{\mathcal{D}}}_{v}$ refers to the set of administrated drug for the condition *v*. Similarly, only the top 50% of the highest-weighted drug-based relations are retained.

### Knowledge-guided domain selection

After constructing the condition KG ${\mathcal{G}}=({\mathcal{V}},{\mathcal{E}})$, condition relationships are embedded into a shared latent space using a KG embedding model. In this study, we adopt the TuckER model^56^. TuckER factorises the KG tensor ${\mathcal{T}}\in {{\mathbb{R}}}^{| {\mathcal{V}}| \times | {\mathcal{R}}| \times | {\mathcal{V}}| }$ into a shared core tensor ${\bf{W}}\in {{\mathbb{R}}}^{d\times d\times d}$ and the embeddings of the relation / entity. The score for a triple (*v*_*i*_, *r*_*k*_, *v*_*j*_) is computed as

5 $${{\bf{T}}}_{ikj}={\bf{W}}{\times }_{1}{{\bf{E}}}_{{v}_{i}}{\times }_{2}{{\bf{R}}}_{{r}_{k}}{\times }_{3}{{\bf{E}}}_{{v}_{j}},$$

where ${{\bf{E}}}_{{v}_{i}},{{\bf{E}}}_{{v}_{j}}\in {{\mathbb{R}}}^{d}$ represent the embeddings of conditions *v*_*i*_ and *v*_*j*_, respectively, ${{\bf{R}}}_{{r}_{k}}\in {{\mathbb{R}}}^{d}$ denotes the embedding of relation *r*_*k*_, and ×_*n*_ indicates the mode-*n* tensor product.

For a target rare condition *v*_*t*_, the most similar source conditions are selected based on the cosine similarity of the condition embeddings:

6 $${{\mathcal{S}}}^{* }=\arg \mathop{\max }\limits_{\begin{array}{l}{{\mathcal{S}}}^{* }\subseteq {\mathcal{S}}\\ | {{\mathcal{S}}}^{* }| =k\end{array}}\mathop{\sum }\limits_{{v}_{s}\in {{\mathcal{S}}}^{* }}\frac{{{\bf{E}}}_{{v}_{t}}\cdot {{\bf{E}}}_{{v}_{s}}}{||{{\bf{E}}}_{{v}_{t}}||||{{\bf{E}}}_{{v}_{s}}||}.$$

The top-*k* conditions with the highest similarity scores are selected as source domains for adaptation.

### Condition-agnostic pre-training

We develop KnowRare using an LSTM network^57^ as the backbone for time-series encoding, given its proven effectiveness in capturing temporal dependencies in sequential EHR data^58^. To capture general temporal and contextual representations from EHR data, the proposed KnowRare framework includes a condition-agnostic pre-training stage, ensuring that the model learns generalisable patterns without overfitting to any specific task. Specifically, we employ a self-supervised method based on next-step prediction.

The encoder consists of two separate modules: a temporal encoder *f*^temp^ for time-series variables **X** and a contextual encoder *f*^cont^ for contextual variables **C**. The encoded variables are concatenated and mapped to obtain the latent representation:

7 $${{\bf{h}}}_{t}={f}^{{\rm{proj}}}\left({f}^{{\rm{temp}}}({{\bf{x}}}_{t}),{f}^{{\rm{cont}}}({\bf{C}})\right),$$

where ${{\bf{h}}}_{t}\in {{\mathbb{R}}}^{{d}_{h}}$ is the hidden representation at time *t*.

By self-supervised learning, the encoder extracts general latent representations that can be quickly adapted to rare conditions. The pre-training objective is designed as a multivariate trajectory reconstruction task, where a decoder *f*^dec^ predicts the next timestep conditioned on the fused representation:

8 $${{\mathcal{L}}}_{\mathrm{pre}-\mathrm{train}}=\frac{1}{T-1}\mathop{\sum }\limits_{t=1}^{T-1}\,{||{{\bf{X}}}_{t+1}-{f}^{\mathrm{dec}}({{\bf{h}}}_{t})||}^{2}.$$

By disentangling latent variables with task-specific patterns, this stage learns condition-agnostic representations that encode general knowledge as the initial parameters for target condition adaptation. This stage is valuable in dealing with data scarcity.

#### Algorithm 1

Training Process of KnowRare

**Require:** EHR data {(**X**_*p*_, **C**_*p*_, *y*_*p*_)}, condition KG ${\mathcal{G}}=({\mathcal{V}},{\mathcal{E}})$, rare condition *v*_*t*_, hyperparameters *k*, *λ*

**Ensure:** Trained model parameters θ^*^

1: **Step 1: Condition-agnostic Pre-training**

2: Initialise encoder parameters *θ*_0_

3: **for** each patient *p* **do**

4: **for** *t* = 1 to *T* − 1 **do**

5: **h**_*t*_ ← *f*^proj^(*f*^temp^(**x**_*t*_), *f*^cont^(**C**_*p*_))

6: $${\widehat{{\bf{X}}}}_{t+1}\leftarrow {f}^{{\rm{dec}}}({{\bf{h}}}_{t})$$

7: Update *θ*_0_ with Equation (8)

8: **end** **for**

9: **end** **for**

10: **Step 2: Knowledge-guided Domain Adaptation**

11: Obtain embeddings {**E**_*v*_} from ${\mathcal{G}}$ with Equation 5

12: Select ${{\mathcal{S}}}^{* }$ of *k* conditions based on {**E**_*v*_} with Equation (6) 13: Initialise *θ* ← *θ*_0_, define discriminator *d*_*ϕ*_

14: **while** not converged **do**

15: Sample mini-batches from ${{\mathcal{S}}}^{* }$ and ${{\mathcal{D}}}_{t}$

16: **h**_*T*_ ← *f*^proj^(*f*^temp^(**X**), *f*^cont^(**C**))

17: $$\widehat{y}\leftarrow {f}_{\theta }({{\bf{h}}}_{T})$$

18: Compute losses $${{\mathcal{L}}}_{{\rm{pred}}},{{\mathcal{L}}}_{{\rm{adv}}}$$

19: Update *θ* and *ϕ* with Equation (10)

20: **end** **while**

21: **return** Model parameters θ^*^ for *v*_*t*_

### Joint adversarial domain adaptation

The final stage of the KnowRare framework employs adversarial learning to align condition-level distributions for robust adaptation to the target rare condition. Leveraging the final time-step latent representation **h**_*T*_ that encodes the full temporal dynamics of the input variables, we mitigate the difference in the joint distribution of the latent representation and the prediction. Unlike prior works that align marginal distributions^59,60^, we hypothesise that domain shifts arise from discrepancies in the joint distribution *P*(**h**_*T*_, *y*) of both representations and clinical outcomes. To address this, we propose a joint adversarial domain adaptation that considers both latent representations and task-specific predictions. Specifically, a joint discriminator *d*_*ϕ*_ operates on the concatenation of **h**_*T*_ and predicted outcome $\widehat{y}$, aligning the variance in the joint distribution:

9 $${{\mathcal{L}}}_{{\rm{adv}}}=-{{\mathbb{E}}}_{({{\bf{h}}}_{T},\widehat{y})}\mathop{\sum }\limits_{i=1}^{| {{\mathcal{S}}}^{* }| +1}{y}_{{{\mathcal{D}}}_{i}}\log {d}_{\phi }({{\bf{h}}}_{T},\widehat{y}),$$

where ${y}_{{{\mathcal{D}}}_{i}}\in {\mathcal{V}}$ denotes the domain labels.

With the discriminator and the adversarial loss, we induce a minimax optimisation process:

10 $$\mathop{\min }\limits_{\theta }\mathop{\max }\limits_{\phi }{{\mathcal{L}}}_{{\rm{adv}}}({{\bf{h}}}_{T},\widehat{y};\theta ,\phi ),$$

where *θ* and *ϕ* denote the encoder and discriminator parameters, respectively. Through this adversarial interaction, the encoder learns to extract domain-invariant representations, while the discriminator continuously refines its ability to distinguish domains. Eventually, the process converges to a Nash equilibrium, where the encoder produces latent representations that minimise domain discrepancy, facilitating effective domain adaptation.

The outcome prediction loss is computed via cross-entropy:

11 $${{\mathcal{L}}}_{{\rm{pred}}}={{\mathcal{L}}}_{{\rm{CE}}}\left({f}_{\theta }({\bf{X}},{\bf{C}}),y\right).$$

To counteract data imbalance across conditions, we apply the inverse propensity score weighting, assigning each sample a weight *w*_*v*_ = 1/*p*(*v*), where *p*(*v*) is the prevalence of the condition *v*. This prioritises underrepresented conditions during training.

The unified objective combines prediction and adversarial losses, with *λ* balancing predictive performance and domain adaptation:

12 $${{\mathcal{L}}}_{{\rm{total}}}={{\mathcal{L}}}_{{\rm{pred}}}+\lambda {{\mathcal{L}}}_{{\rm{adv}}}.$$

For a clearer understanding, Algorithm 1 outlines the overall training process of KnowRare. The process contains two steps: (1) Condition-agnostic pre-training, where the encoder is pre-trained to learn generalisable temporal patterns; (2) Knowledge-guided domain adaptation, where the most similar source conditions are identified, and the model is trained with adversarial learning to align distributions across conditions.

## Supplementary information

Supplementary Information

## Data Availability

The MIMIC-III and eICU databases analysed in this study are available on the PhysioNet repositories https://physionet.org/content/mimiciii/1.4/ and https://physionet.org/content/eicu-crd/2.0/.

## Code availability

The code used for data processing and model development is available at https://github.com/JasonZuu/KnowRare.
