# Supplementary material for: Bridging data gaps of rare conditions in ICU: a multi-disease adaptation approach for clinical prediction
Source: NPJ Digit Med. 2026 Jan 3;9:7. doi: 10.1038/s41746-025-02176-y (PMC12770491; doi:10.1038/s41746-025-02176-y)
Supplement: Supplementary file 1 — Supplementary Information [file 41746_2025_2176_MOESM1_ESM.pdf]

Supplementary Information: Bridging Data Gaps of Rare Conditions in ICU: A Multi-Disease Adaptation Approach for Clinical Prediction

A Visualisation of Challenges of rare conditions in the ICU

We identify two challenges posed by rare conditions in the ICU for DL-based clinical prediction (Figure S1):

- **Data Scarcity**: The low prevalence of rare conditions results in limited samples within the EHR. Figure S1(a) shows that 383 and 192 conditions in MIMIC-III[1] and eICU[2], respectively, have a prevalence of less than 1 in 2,000 cases. The scarcity of data impedes the learning of robust patterns, often resulting in overfitting the model or inadequate generalisation[3,4].

- **intra-condition heterogeneity**: Variability in clinical manifestations, measurements, and outcomes among patients with the same condition[5]. Figure S1(b) Common conditions exhibit a more concentrated distribution, while rare conditions exhibit a notably wider distribution. The inherent diversity of rare conditions challenges the robustness of DL methods, especially with limited training samples[6,7].

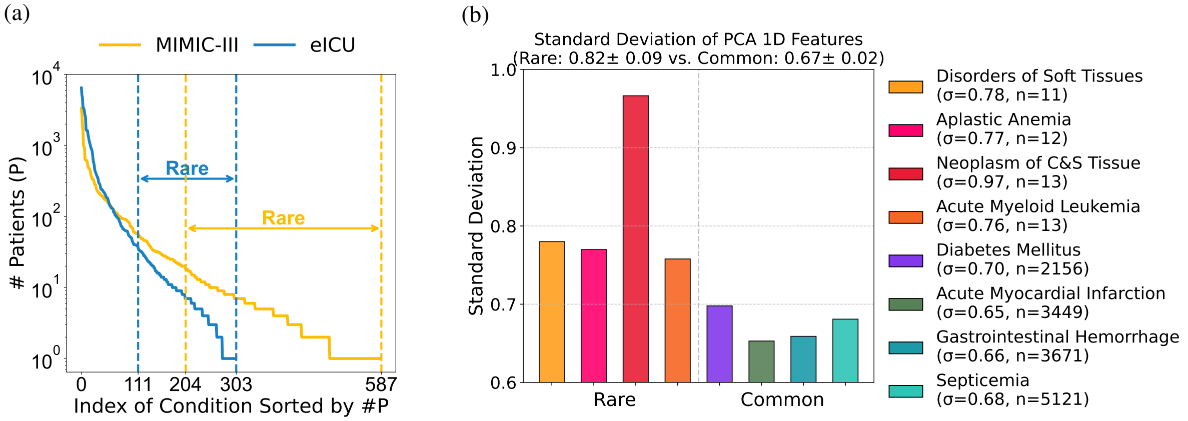

Figure S1. Data scarcity and intra-condition heterogeneity of rare conditions in ICU datasets. (a) Condition prevalence in the MIMIC-III and eICU datasets. Conditions are indexed and labelled as *Rare* if their prevalence is fewer than one case per 2,000 patients. In total, 383 of 587 conditions in MIMIC-III and 192 of 303 conditions in eICU are considered rare, highlighting the significant proportion of rare conditions in the ICU. (b) Histogram of standard deviation (σ) values of one-dimensional variables obtained by applying Principal Component Analysis (PCA) to patient variables for four common and four rare conditions in eICU. Here, n denotes the number of patients for each condition. The mean σ for rare conditions (0.82 ± 0.09) is higher and more variable compared to common conditions (0.67 ± 0.02), illustrating greater intra-condition heterogeneity among rare conditions. Plots are generated using matplotlib (Python).

B Data Processing

We extracted demographic, vital sign, and laboratory test variables from MIMIC-III and eICU, as detailed in the Supplementary Table S1. The data extraction procedure involved several stages, including the selection of patient cohorts, the aggregation of time-series variables, the imputation of missing values, and the normalisation of the data.

We excluded patients with insufficient data availability, specifically, patients with hospital stays shorter than 48 hours in the MIMIC-III dataset and ICU stays shorter than 24 hours in the eICU dataset. Subsequently, we assigned a primary diagnosis to each patient based on the first three digits of their ICD-9-CM codes. Conditions with fewer than ten patients were excluded to ensure adequate sample sizes for robust evaluations. Following these criteria, the resulting dataset included 38,360 patient samples from MIMIC-III and 72,536 from eICU. Each dataset was then partitioned into training, validation, and test subsets using a 4:1:1 ratio, stratifying patients by condition to ensure representative distributions across all splits. After cohort selection, we extracted vital signs and laboratory test measurements from structured EHR databases. For the MIMIC-III dataset, we collected the available measurements within the last 48 hours after hospital admission. For eICU, the data were extracted from the initial 24 hours after admission to the ICU. Demographic information, including age, gender, and race, was obtained directly from patient metadata.

Table S1. Extracted variables from MIMIC-III and eICU

Category	variables	MIMIC-III	eICU	Description
Demographics	Age	✓	✓	The age of the patient at the time of hospital admission.
	Gender	✓	✓	The gender of the patient (Male or Female).
	Race	✓	✓	The racial or ethnic category of the patient.
Vital Sign	Heart Rate	✓	✓	Measures the rate of heartbeats per minute.
	Systolic/Diastolic BP	✓	✓	Measures systolic and diastolic blood pressure.
	Arterial Pressure	✓	✓	Calculates the average blood pressure during one cycle.
	Respiratory Rate	✓	✓	Measures the number of breaths per minute.
	SpO2, Peripheral	✓	✓	Measures blood oxygen saturation levels.
	Temperature	✓	✓	Measures body temperature in Celsius.
	Bedside Glucose	✓	✓	Monitors blood glucose levels.
Lab Test	Albumin	✓	✓	A protein in blood plasma, used to assess liver and kidney function.
	Anion Gap	✓	✓	A measure of ions in the blood, used to detect acid-base imbalances.
	Bands	✓		Immature white blood cells, indicative of infection or inflammation.
	Bicarbonate	✓	✓	A buffer in blood, used to assess acid-base status.
	Bilirubin	✓	✓	A product of red blood cell breakdown. Used to evaluate liver function.
	Blood Urea Nitrogen	✓		Indicates kidney function and hydration status.
	Chloride	✓	✓	An electrolyte, used to assess hydration and acid-base balance.
	Creatinine	✓	✓	A waste product from muscle metabolism.
	Glucose	✓		Blood sugar level.
	Hematocrit	✓	✓	The proportion of red blood cells in the blood.
	Haemoglobin	✓	✓	A protein in red blood cells, carries oxygen throughout the body.
	International Normalised Ratio	✓		Used to evaluate blood coagulation.
	Lactate	✓	✓	Indicates tissue oxygenation and metabolic status.
	Platelet Count	✓	✓	Used to assess blood clotting ability.
	Potassium	✓		An electrolyte, critical for heart and muscle function.
	Prothrombin Time	✓	✓	Measures blood clotting speed, used to monitor anticoagulant therapy.
	Partial Thromboplastin Time	✓	✓	Measures clotting, used to detect bleeding disorders.
	Sodium	✓	✓	An electrolyte, critical for fluid balance and nerve function.
	White Blood Cell Count	✓		Indicates immune response and infection.

[1] A checkmark (✓) indicates that the variable is available and was extracted from the corresponding dataset.

To construct structured time-series representations, the extracted variables were aggregated into consistent temporal intervals, following established benchmark methodologies[8,9]. Specifically, MIMIC-III data were segmented into 24 two-hour intervals during the last 48 hours of admission, calculating the mean values within each interval. Similarly, eICU data were segmented into 24 one-hour intervals that cover the first 24 hours of admission to the ICU. We adopted the settings from two established benchmarks: a two-hour window for 30-day readmission prediction using the last 48 hours of ICU stay[8], and a one-hour window for ICU mortality, LoS, and phenotyping using the first 24 hours of ICU stay[9]. This design ensures fair comparison with prior baseline models. The sensitivity and variability analysis of different time resolutions is presented in Supplementary E. Missing measurements within these intervals initially remained as missing values. To address missing data, we implemented widely accepted imputation techniques[10,11]. Forward imputation was applied for the first time, replacing missing values with the most recent available measurement from the same patient. If forward imputation was not possible due to a lack of previous data, backwards imputation was used, substituting the next available measurement. Any residual missing values after these two steps were filled in with the mean of the corresponding variable in the training set. This approach ensured the independence between the training set and the validation/test sets. The sensitivity analysis of imputation methods is presented in Supplementary F.Lastly, to standardise variable scales across datasets, continuous variables underwent Z-score normalisation using the mean and standard deviation computed exclusively from the training set.

Table S2. Statistics for Selected Rare Conditions in MIMIC-III

Conditions (ICD-9-CM Code Level3)	# Patients	# Hospital Visits	Age	Female Rate	90-Day Mortality	30-Day Readmission Rate
Mycoses (117)	13	13	50.17	0.46	0.23	0.08
Anemia (280)	12	12	67.70	0.42	0.25	0.25
Hodgkin's Disease (201)	12	12	55.44	0.58	0.08	0.08
Neoplasm of Digestive and Respiratory Systems (235)	12	12	68.60	0.67	0.25	0.08
Bronchiectasis (494)	11	11	72.49	0.55	0.18	0.09
Herpetic Whitlow (054)	11	11	51.43	0.36	0.18	0.18
Malignant Neoplasm of Tongue (141)	10	10	60.06	0.30	0.10	0.00
Neoplasms of Unspecified Nature (239)	10	10	64.30	0.30	0.10	0.10
Effects of Reduced Temperature (991)	10	10	59.17	0.30	0.20	0.10
Open Wound of Neck (874)	10	10	43.38	0.30	0.10	0.10

Table S3. Statistics for Selected Rare Conditions in eICU

Condition (ICD-9-CM Code Level3)	# Patients	# ICU Stays	Age	Female Rate	ICU Mortality	Median ICU LoS (Q1-Q3, days)
Meningitis (322)	14	16	64.88	0.57	0.06	2.9 (2.1, 5.4)
Abscess of Lung and Mediastinum (513)	14	14	51.29	0.36	0.07	3.8 (2.0, 5.5)
Acute Myeloid Leukemia (205)	13	13	58.23	0.54	0.23	2.2 (1.6, 4.4)
Acute Pericarditis (420)	12	14	61.00	0.33	0.21	2.0 (1.3, 2.9)
Malignant Neoplasm of Connective and Other Soft Tissue (171)	11	13	63.08	0.18	0.23	2.9 (1.9, 3.7)
Aplastic Anemia (284)	11	12	61.75	0.64	0.08	1.9 (1.5, 3.1)
Effects of Reduced Temperature (991)	11	11	62.45	0.45	0.09	2.7 (1.6, 3.4)
Other Disorders of Soft Tissues (729)	10	11	62.09	0.70	0.09	2.0 (1.4, 2.7)
Malignant Neoplasm of Gallbladder and Extra-hepatic Bile Ducts (156)	10	11	52.91	0.60	0.09	1.7 (1.3, 2.3)
Fracture of Base of Skull (801)	10	10	51.60	0.50	0.20	2.7 (1.5, 3.6)

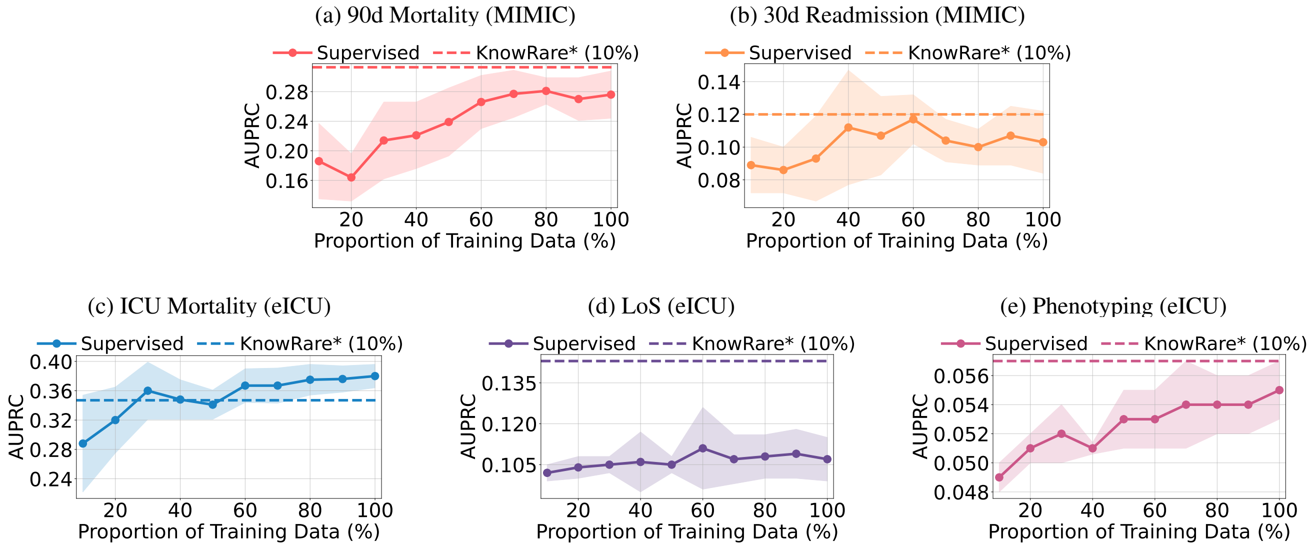

Figure S2. Generalisation to common conditions under limited-data scenarios with Pre-training. Evaluating KnowRare's robustness by training it with only 10% of the available septicemia data, while the LSTM baseline model uses septicemia data ranging from 10% to 100%. Prediction tasks include: (a) 90-day mortality prediction after hospital discharge (MIMIC-III), (b) 30-day readmission prediction after hospital discharge (MIMIC-III), (c) ICU mortality prediction (eICU), (d) Remaining length of stay prediction (eICU), and (e) Phenotyping prediction (eICU). Points represent mean values, and shaded regions indicate standard deviation over five runs. * refers to KnowRare with the condition-agnostic pre-trained module. Plots are generated using matplotlib (Python).

C Evaluation of KnowRare's Applicability with Pre-training

This experiment evaluated whether enabling the condition-agnostic pre-training module affects KnowRare's applicability in limited-data scenarios. We tested KnowRare on septicaemia with the training set restricted to 10% of the available samples while allowing condition-agnostic pre-training. The supervised baseline was a standard LSTM model.

As shown in Figure S2, the results were consistent with our main findings. For 90-day mortality, 30-day readmission, remaining LoS, and phenotyping, KnowRare with pre-training outperformed the baseline LSTM even when the latter was trained on 100% of the data. In ICU mortality, KnowRare did not fully exceed a trained LSTM but still outperformed the model trained on 50% of the data. These results confirm the effectiveness of KnowRare in leveraging transferable clinical knowledge and demonstrate that the framework remains robust in both pre-trained and non-pretrained settings.

D Analysis of ICD-9-CM Code Levels

This experiment evaluated the impact of different levels of ICD-9-CM codes on sample size distributions of conditions. We compared the original full-length ICD-9-CM codes with Level 4 and Level 3 codes, where higher-level aggregation groups diagnosed conditions into broader categories. Aggregation above Level 3 is not meaningful, as ICD-9-CM already defines broad condition categories at this level.

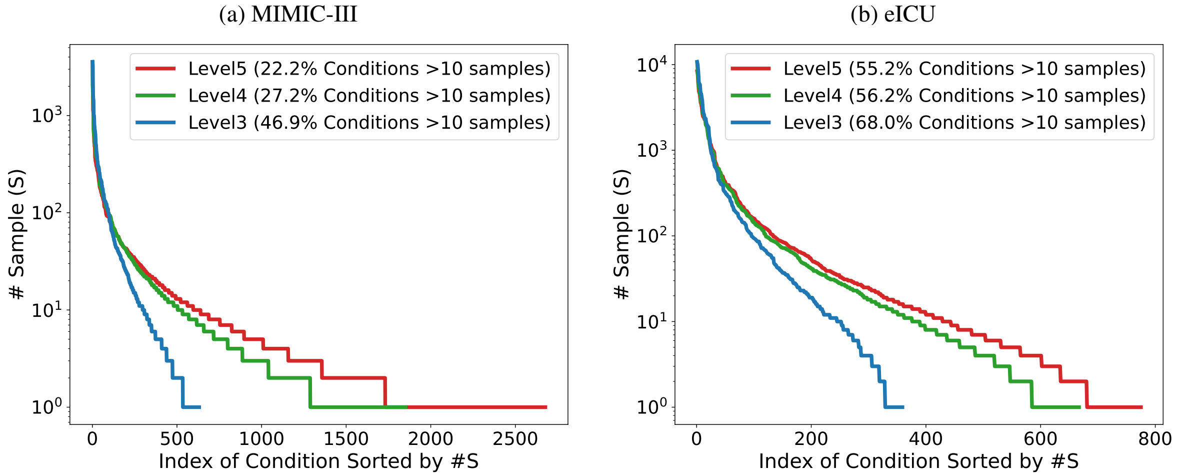

Figure S3. Condition sample distributions under different ICD-9-CM levels in MIMIC-III and eICU. Plots are generated using matplotlib (Python).

As shown in Figures S3, aggregating at higher levels substantially reduced data sparsity. In MIMIC-III, only 22.2% of conditions with original codes had more than 10 samples, compared with 27.2% at Level 4 and 46.9% at Level 3. A similar trend was observed in eICU, where the proportions increased from 55.2% (original) and 56.2% (Level 4) to 68.0% at Level 3. These results confirm that aggregation at the Level 3 level provides a better balance between clinical specificity and sample sufficiency, making it a suitable choice for downstream modelling tasks.

E Sensitivity and Variability Analysis of Time Resolution

To assess the impact of temporal resolution on model performance, we conducted a sensitivity analysis using time windows of 30 minutes, 1 hour, and 2 hours. Specifically, we repeated the data processing steps and hyperparameter training process with the same settings as in the main experiments, and trained the KnowRare on five different random seeds. As shown in Figures S4(a)(b), in MIMIC-III, the 30-minute and 2-hour resolutions yielded similar performance, while the 1-hour resolution consistently underperformed. In contrast, for eICU, the 1-hour resolution achieved the best performance, outperforming both 30-minute and 2-hour settings. These results indicate that the optimal time resolution is dataset dependent, shaped by factors such as recording frequency, data completeness, and inter-institutional variability. The sensitivity of time resolution in clinical prediction for rare conditions reveals interesting patterns, and we will explore its effects further in future work.

We further evaluated whether changing the time resolution altered the variability of time-series features. Specifically, we computed the coefficient of variation (CV) within each time window for all features and reported the average and standard deviation across patients. As shown in Figures S4(c)(d), the CV distributions were similar across time regardless of the time resolution used. This indicates that the choice of time resolution does not change the variability of the data, and that performance differences observed in the sensitivity study are more likely due to differences in data sparsity and information density across time resolutions rather than changes in feature variability.

F Sensitivity Analysis of Imputation Method

To evaluate the effect of different imputation strategies on model performance, we conducted a sensitivity analysis comparing LOCF/NOCB, global mean imputation[12], and linear interpolation[12] for missing value imputation. We repeated the same data processing steps and hyperparameter training process as in the main experiments, and trained KnowRare with five different random seeds for each setting.

As shown in Figures S5(a)(b), LOCF/NOCB consistently achieved the best performance across prediction tasks in both MIMIC-III and eICU, significantly outperforming mean imputation and interpolation on all tasks. These findings are consistent with observations from previous studies on common-condition prediction tasks, where LOCF/NOCB has been shown to better preserve temporal structure in sparse clinical time-series data[13]. This analysis confirms that LOCF/NOCB is the most effective imputation strategy for rare-condition prediction tasks.

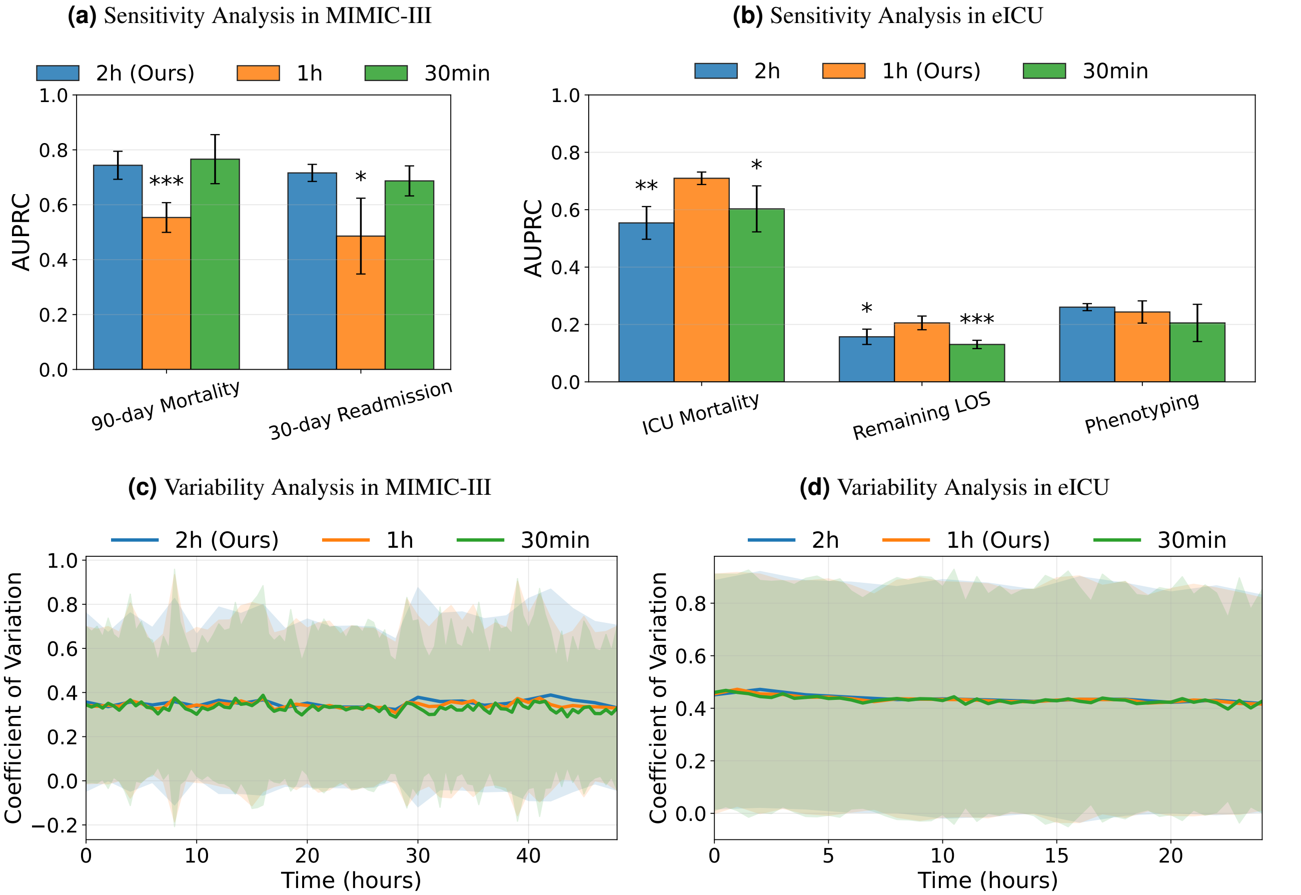

Figure S4. Sensitivity and variability analyses across different time resolutions (30 minutes, 1 hour, 2 hours) in MIMIC-III and eICU.(a) Sensitivity analysis in MIMIC-III, with t-tests showing significant differences for 1 h and 30 min compared with the adopted 2 h resolution.(b) Sensitivity analysis in eICU, with t-tests showing significant differences for 2 h and 30 min compared with the adopted 1 h resolution. (c–d) Variability analysis of features across time windows. Lines indicate the mean CV across features, and shaded areas represent the standard deviation of CV. Plots are generated using matplotlib (Python).

G Discrimination Analysis of KnowRare

To further evaluate the practical utility of KnowRare in real-world clinical settings, we extend our analysis by examining model discrimination across different thresholds. Since all tasks are severely class-imbalanced in rare conditions, AUPRC is particularly important, where AUROC alone may be misleading. Also, our model was optimised on AUPRC using the validation set. To provide a complete view, we report both Receiver Operating Characteristic (ROC) and Precision–Recall (PR) curves for each method. ROC curves illustrate the trade-off between false positives and false negatives, while PR curves highlight performance on the minority (positive) class, which is critical in imbalanced clinical datasets. For comparison, we also evaluated three baseline methods: (i) a standard LSTM[14], which serves as the backbone of KnowRare; (ii) RareMed[15], the latest few-shot clinical prediction method; and (iii) MANYDG[16], the latest domain adaptation-based method for clinical prediction. As shown in Figures S6 and S7, KnowRare demonstrates superior or similar ROC and PRC profiles compared to the baselines.

H Calibration Analysis of KnowRare

To assess whether KnowRare's predicted probabilities can be reliably interpreted as outcome risks, we conducted a calibration analysis using two standard methods: temperature scaling[17] and isotonic regression[18]. For temperature scaling, the parameter was optimised by minimising the negative log-likelihood (NLL) within the range 0.2–5.0. Calibration performance was evaluated using Expected Calibration Error (ECE) and NLL.

As shown in the reliability diagrams (Figures S8), calibration is necessary to improve the reliability of predicted probabilities,

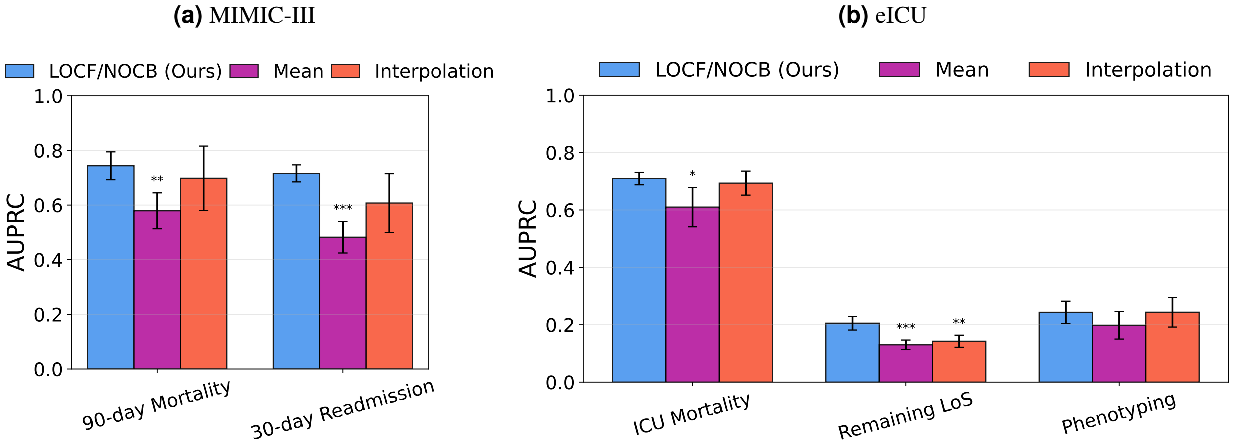

Figure S5. Sensitivity analyses across different time resolutions (30 minutes, 1 hour, 2 hours) in MIMIC-III and eICU.(a) Sensitivity analysis in MIMIC-III, with t-tests showing significant differences for 1 h and 30 min compared with the adopted 2 h resolution.(b) Sensitivity analysis in eICU, with t-tests showing significant differences for 2 h and 30 min compared with the adopted 1 h resolution. Plots are generated using matplotlib (Python).

but it remains challenging in rare conditions. In particular, for 30-day readmission and ICU mortality prediction, both temperature scaling and isotonic regression failed to improve either ECE or NLL compared with the uncalibrated KnowRare. This failure is likely due to the mismatch between calibration tuned on the validation set of all conditions and evaluation on the rare-condition test sets. These results highlight the difficulty of transferring calibration parameters to the heterogeneous cohorts of rare conditions.

These findings underscore that while calibration is critical for clinical prediction in rare conditions, it is complicated by the discrepancy between general validation data and rare-condition test distributions. Developing methods to achieve robust calibration without relying on rare-condition labels represents a valuable future research direction.

I Implementation Details

I.1 Model Architecture

We developed the KnowRare framework and all model-agnostic baseline methods using the LSTM as the backbone for encoding time-series data. Specifically, the LSTM encoder consisted of a single-layer network with a hidden dimension of 128. In addition to time-series inputs, demographic variables (age, gender, and race) were processed through a two-layer Multilayer Perceptron (MLP) with LeakyReLU activation functions, also having a hidden dimension of 128. The resulting demographic embeddings were then concatenated with the final hidden state of the LSTM encoder, forming the latent representation. Subsequently, this combined representation was fed into a two-layer MLP classifier to predict clinical outcomes. Other modules of the KnowRare framework, including the decoder used during pre-training and the discriminator used for domain adaptation, were similarly implemented as two-layer MLP networks.

I.2 Hyperparameters

The hyperparameters of all baseline methods are determined using Bayesian optimisation with 30 iterations based on their performance on the validation set. Baseline models that include a pre-training stage (FADA, AdvDiag, SMART, RareMed, and KnowRare) are pre-trained on the entire training set. For KnowRare, we select 10% of all conditions for joint adversarial domain adaptation. All methods are trained for a maximum of 100 epochs, with early stopping applied if no performance improvement is observed for 10 consecutive epochs. We repeated our experiment five times with different random seeds, reporting the mean and standard deviation (std) for each evaluation metric. The hyperparameters for KnowRare and each baseline method are detailed in Table S4. We utilised the Weights & Biases platform to perform an extensive hyperparameter search. The optimal hyperparameters were selected based on the highest AUPRC achieved in the validation set.

I.3 Training Settings

We employ the Adam optimiser for training, initialising with a warm-up phase of a fixed learning rate of 10 epochs. Following this, we apply an exponential decay to the learning rate at a rate of 0.95 after each subsequent epoch. To prevent overfitting and ensure efficient training, we implement an early stopping mechanism that stops training if there is no improvement in validation

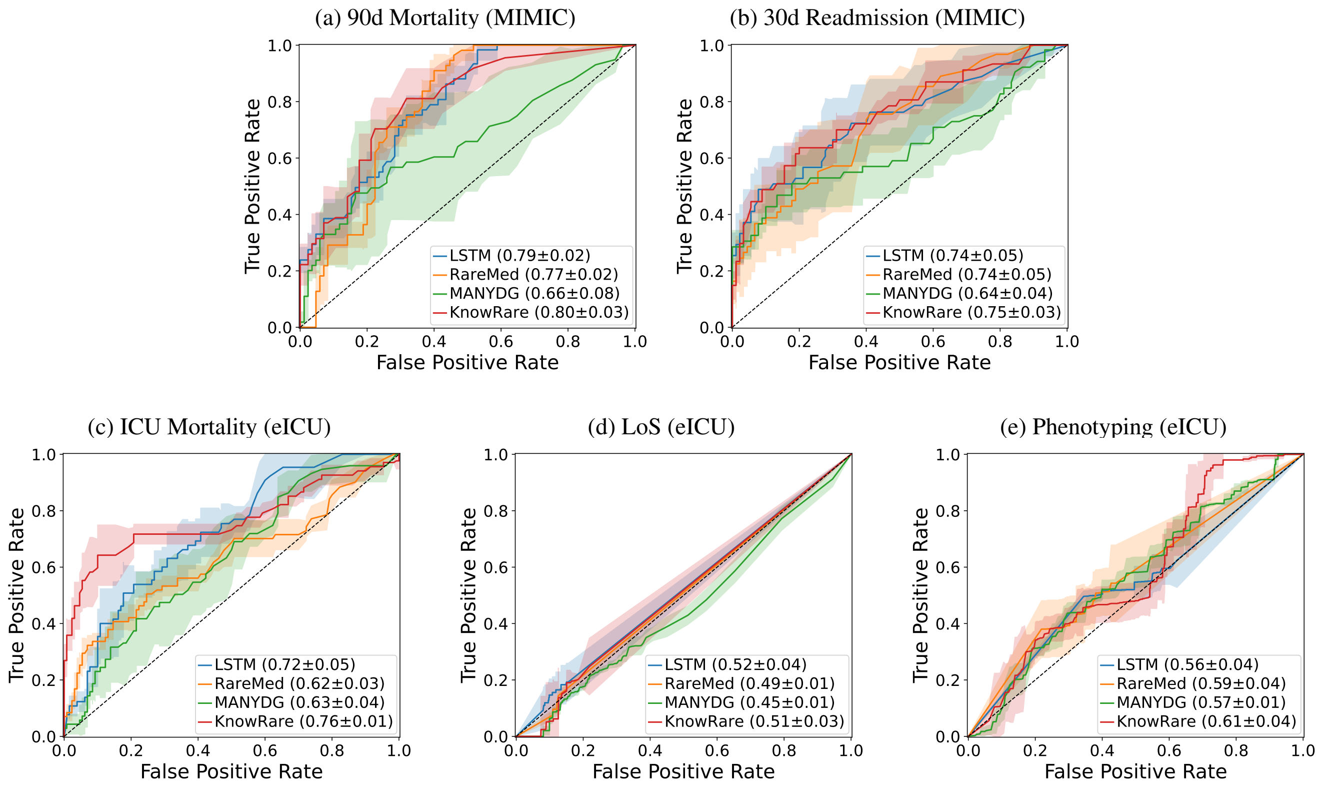

Figure S6. **ROC curves for KnowRare and baseline methods.** Tasks include: (a) 90-day mortality after hospital discharge (MIMIC-III), (b) 30-day readmission after hospital discharge (MIMIC-III), (c) ICU mortality (eICU), (d) remaining length of stay (eICU), and (e) phenotyping (eICU). Curves show the mean over five runs, with shaded areas representing the standard deviation. Plots are generated using matplotlib (Python).

performance over 10 consecutive epochs. The optimiser is configured with $\beta_1 = 0.9$, $\beta_2 = 0.999$, and $\varepsilon = 10^{-8}$, adhering to the default parameters settings.

J Results of knowledge-guided domain selection

To evaluate the categorical relationship between selected source conditions and their target rare condition, we summarised the distribution of source condition categories across MIMIC-III and eICU. As shown in Table S5, the source conditions rarely belonged to the same ICD-9-CM category as the target. On average, only 19.2% of source conditions in MIMIC-III and 9.5% in eICU were in the same category. The vast majority (80.8% for MIMIC-III and 90.5% for eICU, respectively) originated from categories outside of the target's ICD-9-CM category. Table S6 presents the selected source conditions corresponding to the target rare conditions, as identified through the proposed knowledge-guided domain selection method.

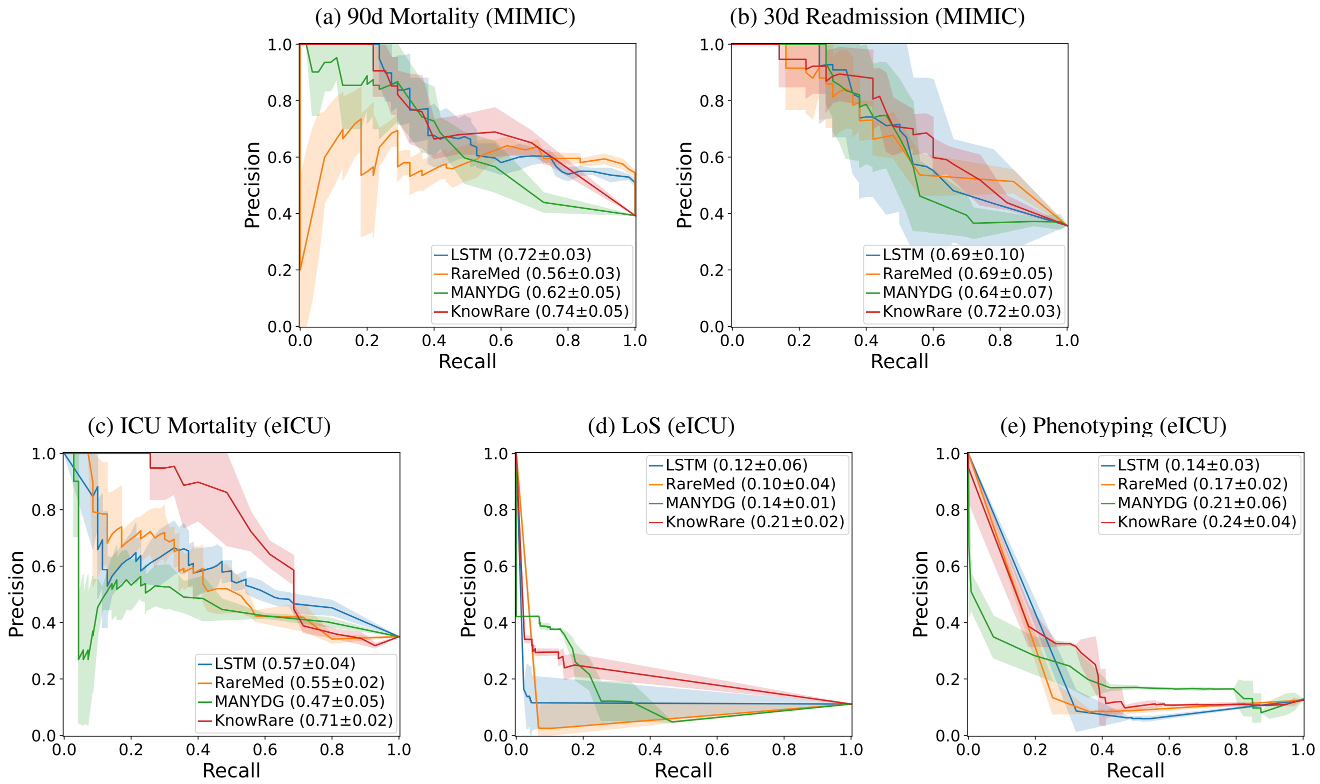

Figure S7. PR curves for KnowRare and baseline methods. Tasks include: (a) 90-day mortality after hospital discharge (MIMIC-III), (b) 30-day readmission after hospital discharge (MIMIC-III), (c) ICU mortality (eICU), (d) remaining length of stay (eICU), and (e) phenotyping (eICU). Curves show the mean over five runs, with shaded areas representing the standard deviation. Plots are generated using matplotlib (Python).

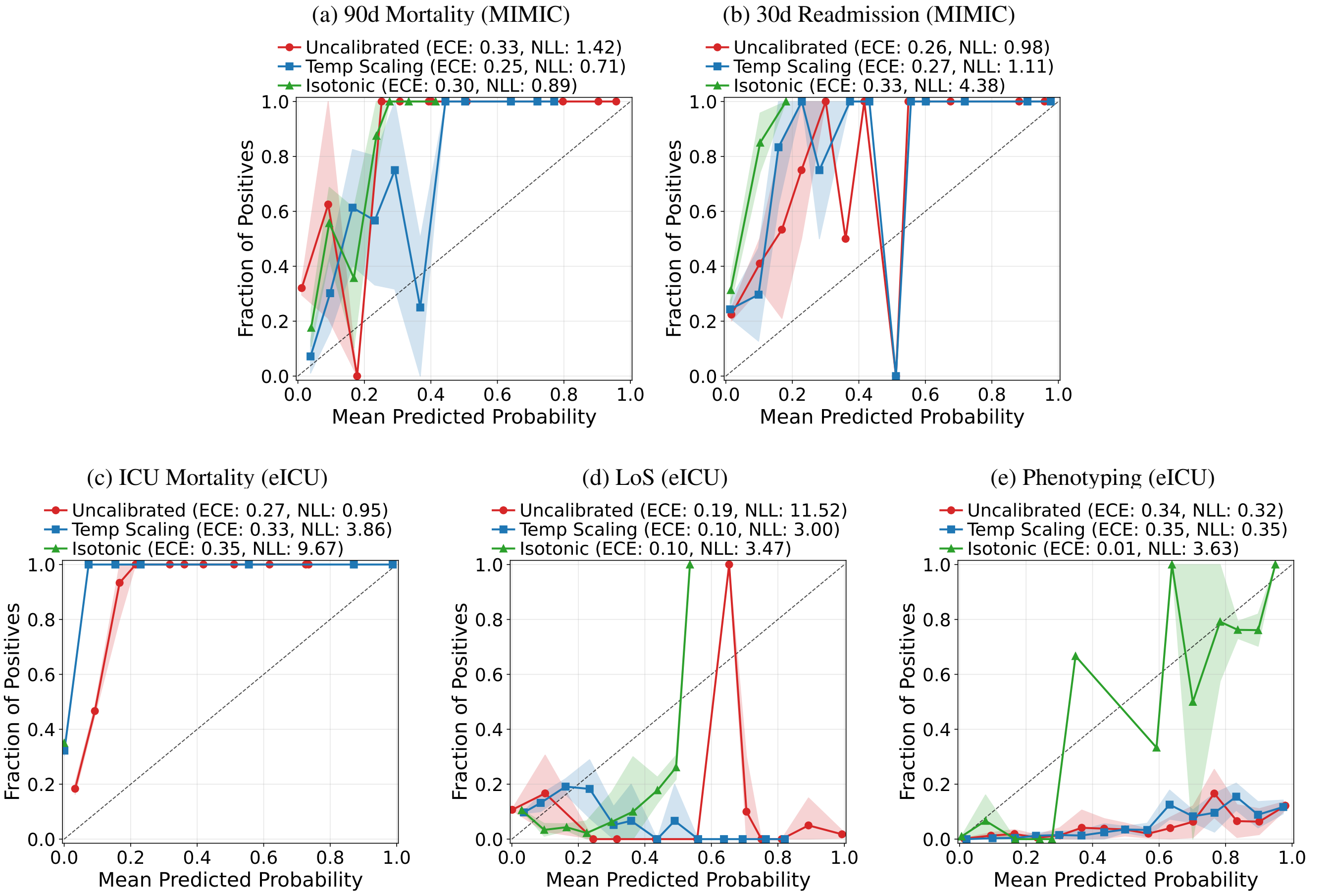

Figure S8. Calibration analysis of KnowRare. Reliability diagrams for (a) 90-day mortality after hospital discharge (MIMIC-III), (b) 30-day readmission after hospital discharge (MIMIC-III), (c) ICU mortality (eICU), (d) remaining length of stay (eICU), and (e) phenotyping (eICU). Calibration methods include temperature scaling (Temp Scaling) and isotonic regression. Each curve represents the mean over five runs, with shaded areas indicating the standard deviation. Reported values show the average ECE and NLL across five random seeds. Plots are generated using matplotlib (Python).

Table S4. Hyperparameter Search Space for Different Methods

Method	Hyperparameter	Search Space
LSTM	Learning Rate	Uniform(1e-5, 1e-3)
	Batch Size	{16, 32, 64, 128}
Transformer	Learning Rate	Uniform(1e-5, 1e-3)
	Batch Size	{16, 32, 64, 128}
RETAIN	Learning Rate	Uniform(1e-5, 1e-3)
	Batch Size	{16, 32, 64, 128}
MetaPred	Meta Learning Rate	Uniform(1e-5, 1e-3)
	Inner Learning Rate	Uniform(1e-5, 1e-3)
	Batch Size	{16, 32, 64, 128}
	Inner Loss Coefficient	{0.1, 1.0, 10.0}
RareMed	Learning Rate	Uniform(1e-5, 1e-3)
	Batch Size	{16, 32, 64, 128}
SMART	Learning Rate	Uniform(1e-5, 1e-3)
	Batch Size	{16, 32, 64, 128}
Stable-CRP	Learning Rate	Uniform(1e-5, 1e-3)
	Batch Size	{16, 32, 64, 128}
FADA	Learning Rate	Uniform(1e-5, 1e-3)
	Discriminator Learning Rate	Uniform(1e-5, 1e-3)
	Batch Size	{16, 32, 64, 128}
	Adversarial Loss Coefficient	{0.1, 0.5, 1.0, 2.0, 10.0}
	Discriminator Update Frequency	Uniform(1, 10)
AdvDiag	Learning Rate	Uniform(1e-5, 1e-3)
	Discriminator Learning Rate	Uniform(1e-5, 1e-3)
	Batch Size	{16, 32, 64, 128}
	Adversarial Loss Coefficient	{0.1, 0.5, 1.0, 2.0, 10.0}
	Discriminator Update Frequency	Uniform(1, 10)
MANYDG	Learning Rate	Uniform(1e-5, 1e-3)
	Batch Size	{16, 32, 64, 128}
KnowRare	Learning Rate	Uniform(1e-5, 1e-3)
	Discriminator Learning Rate	Uniform(1e-5, 1e-3)
	Batch Size	{16, 32, 64, 128}
	Adversarial Loss Coefficient	{0.005, 0.01, 0.02, 0.1}
	Discriminator Update Frequency	Uniform(1, 5)

Table S5. Comparison of Source Condition ICD-9 Categories for MIMIC-III and eICU Datasets

(a) MIMIC-III

Target Rare Condition (ICD-9-CM)	Target Category (ICD-9-CM Chapter)	% Same Category	% Other Categories
Mycoses (117)	Infectious (001-139)	0.0%	100.0%
Iron Deficiency Anemia (280)	Blood (280-289)	3.7%	96.3%
Hodgkin's Disease (201)	Neoplasms (140-239)	14.8%	85.2%
Neoplasm of Digestive/Resp (235)	Neoplasms (140-239)	33.3%	66.7%
Bronchiectasis (494)	Respiratory (460-519)	14.8%	85.2%
Herpetic Whitlow (054)	Infectious (001-139)	0.0%	100.0%
Malignant Neoplasm of Tongue (141)	Neoplasms (140-239)	33.3%	66.7%
Neoplasms of Unspecified Nature (239)	Neoplasms (140-239)	25.9%	74.1%
Effects of Reduced Temperature (991)	Injury (800-999)	29.6%	70.4%
Open Wound of Neck (874)	Injury (800-999)	37.0%	63.0%
**Average**	-	**19.2%**	**80.8%**

(b) eICU

Target Rare Condition (ICD-9-CM)	Target Category (ICD-9-CM Chapter)	% Same Category	% Other Categories
Fracture of Base of Skull (801)	Injury (800-999)	15.8%	84.2%
Other Disorders of Soft Tissues (729)	Musculoskeletal (710-739)	0.0%	100.0%
Malignant Neoplasm of Gallbladder (156)	Neoplasms (140-239)	11.1%	88.9%
Malignant Neoplasm of Conn. Tissue (171)	Neoplasms (140-239)	10.5%	89.5%
Aplastic Anaemia (284)	Blood (280-289)	5.30%	94.70%
Effects of Reduced Temperature (991)	Injury (800-999)	26.3%	73.7%
Acute Pericarditis (420)	Circulatory (390-459)	15.8%	84.2%
Acute Myeloid Leukemia (205)	Neoplasms (140-239)	0.0%	100.0%
Meningitis (322)	Nervous System (320-389)	0.0%	100.0%
Abscess of Lung and Mediastinum (513)	Respiratory (460-519)	10.5%	89.5%
**Average**	-	**9.5%**	**90.5%**

Table S6. The selected source conditions for each rare condition in MIMIC-III and eICU

Rare Condition (ICD-9-CM Code Level 3)	Dataset	Source Conditions (ICD-9-CM Code Level 3)
Mycoses (117)	MIMIC-III	Chronic airway obstruction (510), Other diseases of lung (724), Other diseases of blood and blood-forming organs (289), Other diseases of arteries, arterioles, and capillaries (456), Other disorders of circulatory system (447), Congenital anomalies of heart (745), Other diseases of bone and cartilage (721), Malignant neoplasm of other and unspecified sites (196), Chronic liver disease and cirrhosis (570), Migraine (346), Gastritis and duodenitis (535), Other diseases of blood and blood-forming organs (572), Other diseases of lung (420), Other disorders of circulatory system (437), Other diseases of blood and blood-forming organs (292), Congenital anomalies of heart (746), Other diseases of lung (873), Other diseases of veins and lymphatics, and other diseases of circulatory system (455), Other diseases of lung (710), Other diseases of blood and blood-forming organs (202), Other disorders of circulatory system (444), Other diseases of blood and blood-forming organs (255), Other diseases of blood and blood-forming organs (324), Other diseases of genitourinary system (593), Chronic airway obstruction (494), Gastric ulcer (430), Gastric ulcer (531)
Iron Deficiency Anemia (280)	MIMIC-III	Other diseases of blood and blood-forming organs (507), Epilepsy (345), Other diseases of nervous system (349), Other diseases of respiratory system (519), Chronic ulcer of skin (707), Other diseases of genitourinary system (511), Chronic liver disease and cirrhosis (570), Intestinal infection due to other organisms (008), Conduction disorders (426), Other diseases of blood and blood-forming organs (572), Acute myocardial infarction (410), Alcohol dependence syndrome (303), Other diseases of circulatory system (459), Pneumonia, organism unspecified (482), Other disorders of intestine (569), Symptoms involving respiratory system and other chest symptoms (786), Other diseases of veins and lymphatics, and other diseases of circulatory system (453), Endocarditis (424), Other forms of heart disease (440), Cardiomyopathy (425), Other diseases of blood and blood-forming organs (288), Other diseases of genitourinary system (593), Osteoarthrosis and allied disorders (715), Gastrointestinal hemorrhage (578), Other disorders of bone and cartilage (733), Other disorders of circulatory system (443), Other forms of heart disease (411)
Hodgkin's Disease (201)	MIMIC-III	Gastritis and duodenitis (534), Meningitis due to other organisms (047), Other diseases of bone and cartilage (803), Spina bifida (742), Poisoning by antibiotics (980), Injury to blood vessels of thorax (901), Other diseases of blood and blood-forming organs (862), Pneumonia, organism unspecified (480), Other diseases of synovium, tendon, and bursa (711), Acute pharyngitis (464), Other unspecified infectious and parasitic diseases (991), Poisoning by other central nervous system depressants and anesthetics (972), Other diseases of digestive system (212), Intestinal infection due to other organisms (009), Other diseases of heart (404), Diseases of mitral and aortic valves (394), Other unspecified infectious and parasitic diseases (994), Open wound of other and unspecified sites, except limbs (874), Hypertensive heart disease (402), Carcinoma in situ of breast and genitourinary system (233), Inguinal hernia (550), Anomalies of peripheral vascular system (747), Meningitis due to other organisms (136), Poisoning by agents primarily affecting blood constituents (966), Malignant neoplasm of other and unspecified sites (183), Malignant neoplasm of other and unspecified sites (164), Diseases of mitral and aortic valves (395)

continued on next page

Rare Condition (ICD-9-CM Code Level 3)	Dataset	Source Conditions (ICD-9-CM Code Level 3)
Neoplasm of Digestive and Respiratory Systems (235)	MIMIC-III	Other diseases of male genital organs (608), Other diseases of bone and cartilage (803), Meningitis due to other organisms (047), Malignant neoplasm of other and unspecified sites (171), Open wound of other and unspecified sites, except limbs (881), Malignant neoplasm of other and unspecified sites (158), Malignant neoplasm of other and unspecified sites (161), Other diseases of blood and blood-forming organs (862), Pneumonia, organism unspecified (480), Malignant neoplasm of other and unspecified sites (152), Acute pharyngitis (464), Spina bifida (738), Poisoning by other central nervous system stimulants (982), Injury to blood vessels of abdomen (864), Other diseases of heart (404), Intestinal infection due to other organisms (009), Other diseases of blood and blood-forming organs (866), Other diseases of respiratory system (513), Injury to blood vessels of abdomen (902), Other diseases of blood and blood-forming organs (200), Open wound of other and unspecified sites, except limbs (874), Other diseases of blood and blood-forming organs (228), Other diseases of blood and blood-forming organs (209), Other diseases of blood and blood-forming organs (674), Malignant neoplasm of lip,, oral cavity, and pharynx (141), Malignant neoplasm of other and unspecified sites (183), Injury to blood vessels of head and neck (854)
Bronchiectasis (494)	MIMIC-III	Other diseases of male genital organs (608), Rheumatic mitral valve disease (398), Pneumonia due to other specified bacteria (481), Other disorders of circulatory system (447), Malignant neoplasm of pancreas (157), Other alveolar and parietoalveolar pneumonopathies (516), Infection of kidney (590), Fracture of neck of femur (820), Fracture of radius and ulna (813), Fracture of lumbar spine and pelvis (806), Lymphoid leukemia (204), Pneumonia, organism unspecified (480), Osteoarthrosis and allied disorders (711), Multiple myeloma and immunoproliferative neoplasms (203), Malignant neoplasm of kidney and other unspecified urinary organs (189), Congenital anomalies of heart (746), Benign neoplasm of uterus (225), Systemic lupus erythematosus (710), Abscess of lung and mediastinum (513), Gastric ulcer (432), Other diseases of male genital organs (235), Gastric ulcer (430), Anomalies of peripheral vascular system (747), Polyarteritis nodosa and allied conditions (446), Fracture of base of skull (801), Disorders of lipoid metabolism (277), Transient cerebral ischemia (435)
Herpetic Whitlow (054)	MIMIC-III	Empyema (510), Other disorders of soft tissue (728), Osteomyelitis, periostitis, and other infections involving bone (730), Other diseases of arteries, arterioles, and capillaries (456), Congenital anomalies of heart (745), Chronic bronchitis (491), Disorders of adrenal glands (253), Regional enteritis (555), Internal injury of chest, abdomen, and pelvis (860), Chronic liver disease and cirrhosis (570), Migraine (346), Gastritis and duodenitis (535), Acute vascular insufficiency of intestine (557), Acute pericarditis (420), Toxic effect of other substances, chiefly nonmedicinal as to source (292), Internal injury of chest, abdomen, and pelvis (861), Ill-defined descriptions and complications of heart disease (429), Hemorrhoids (455), Other disorders of arteries, arterioles, and capillaries (442), Other diseases of blood and blood-forming organs (202), Epistaxis (784), Other disorders of circulatory system (444), Disorders of adrenal glands (255), Gastric ulcer (430), Poisoning by analgesics, antipyretics, and antirheumatics (965), Neoplasms of uncertain behavior (238), Diseases of pericardium (397)
Malignant neoplasm of Tongue (141)	MIMIC-III	Gastritis and duodenitis (534), Other specified diseases due to viruses (088), Spina bifida (742), Open wound of other and unspecified sites, except limbs (881), Poisoning by antibiotics (980), Malignant neoplasm of other and unspecified sites (158), Malignant neoplasm of other and unspecified sites (161), Other diseases of blood and blood-forming organs (862), Pneumonia, organism unspecified (480), Malignant neoplasm of other and unspecified sites (152), Acute pharyngitis (464), Spina bifida (738), Poisoning by other central nervous system stimulants (982), Poisoning by other central nervous system depressants and anesthetics (972), Poisoning by agents primarily affecting blood constituents (962), Other diseases of heart (404), Other diseases of blood and blood-forming organs (866), Other diseases of blood and blood-forming organs (200), Open wound of other and unspecified sites, except limbs (874), Trigeminal nerve disorders (350), Other diseases of blood and blood-forming organs (209), Other diseases of blood and blood-forming organs (228), Other diseases of male genital organs (235), Other diseases of blood and blood-forming organs (674), Malignant neoplasm of other and unspecified sites (164), Malignant neoplasm of other and unspecified sites (183), Injury to blood vessels of head and neck (854)

continued on next page

Rare Condition (ICD-9-CM Code Level 3)	Dataset	Source Conditions (ICD-9-CM Code Level 3)
Neoplasms of Unspecified Nature (239)	MIMIC-III	Gastritis and duodenitis (534), Meningitis due to other organisms (047), Anomalies of digestive system (751), Injury to blood vessels of thorax (901), Malignant neoplasm of brain (191), Injury to blood vessels of head and neck (851), Malignant neoplasm of liver and intrahepatic bile ducts (156), Other diseases of blood and blood-forming organs (862), Pneumonia, organism unspecified (480), Acute pharyngitis (464), Malignant neoplasm of kidney and other unspecified urinary organs (189), Poisoning by other central nervous system stimulants (982), Poisoning by other central nervous system depressants and anesthetics (972), Neoplasms of uncertain behavior (237), Other diseases of digestive system (212), Malignant neoplasm of other and unspecified sites (193), Other diseases of heart (404), Injury to blood vessels of abdomen (902), Other unspecified infectious and parasitic diseases (994), Encephalitis, myelitis, and encephalomyelitis (323), Hypertensive heart disease (402), Carcinoma in situ of breast and genitourinary system (233), Spinal cord injury without evidence of spinal bone injury (952), Poisoning by other agents primarily affecting blood constituents (970), Anomalies of peripheral vascular system (747), Meningitis due to other organisms (136), Polyarteritis nodosa and allied conditions (446)
Effects of Reduced Temperature (991)	MIMIC-III	Meningitis due to other organisms (047), Other diseases of bone and cartilage (803), Spina bifida (742), Open wound of other and unspecified sites, except limbs (881), Poisoning by antibiotics (980), Fracture of ankle (825), Malignant neoplasm of breast (174), Anomalies of digestive system (751), Injury to blood vessels of thorax (901), Malignant neoplasm of brain (191), Other diseases of blood and blood-forming organs (862), Poisoning by other central nervous system stimulants (982), Other diseases of blood and blood-forming organs (201), Other diseases of digestive system (212), Poisoning by agents primarily affecting blood constituents (962), Intestinal infection due to other organisms (009), Other diseases of heart (404), Diseases of mitral and aortic valves (394), Coagulation defects (666), Hypertensive heart disease (402), Trigeminal nerve disorders (350), Other diseases of blood and blood-forming organs (228), Acute appendicitis (540), Anomalies of peripheral vascular system (747), Meningitis due to other organisms (136), Malignant neoplasm of lip, oral cavity, and pharynx (141), Malignant neoplasm of other and unspecified sites (183)
Open Wound of Neck (874)	MIMIC-III	Other diseases of bone and cartilage (803), Other inflammatory conditions of skin (088), Spina bifida (742), Open wound of other and unspecified sites, except limbs (881), Poisoning by antibiotics (980), Malignant neoplasm of other and unspecified sites (161), Other diseases of blood and blood-forming organs (862), Pneumonia, organism unspecified (480), Malignant neoplasm of other and unspecified sites (152), Acute pharyngitis (464), Poisoning by other central nervous system stimulants (982), Poisoning by other central nervous system depressants and anesthetics (972), Poisoning by agents primarily affecting the cardiovascular system (962), Other diseases of heart (404), Intestinal infection due to other organisms (009), Other diseases of blood and blood-forming organs (866), Other unspecified infectious and parasitic diseases (994), Diseases of mitral and aortic valves (394), Delivery in a completely normal case (666), Trigeminal nerve disorders (350), Other diseases of blood and blood-forming organs (209), Other diseases of blood and blood-forming organs (228), Other diseases of blood and blood-forming organs (674), Malignant neoplasm of lip, oral cavity, and pharynx (141), Malignant neoplasm of other and unspecified sites (164), Malignant neoplasm of other and unspecified sites (183), Injury to blood vessels of head and neck (854)

continued on next page

Rare Condition (ICD-9-CM Code Level 3)	Dataset	Source Conditions (ICD-9-CM Code Level 3)
Fracture of Base of Skull (801)	eICU	Empyema (510), Other disorders of soft tissue (728), Poisoning by other specified drugs and medicinal substances (968), Pancreatic disorders (576), Malignant neoplasm of rectum, rectosigmoid junction, and anus (154), Diseases of hard tissues of teeth (523), Other vascular insufficiencies of intestine (557), Toxic effect of other substances, chiefly nonmedicinal as to source (292), Fracture of shaft of femur (821), Secondary malignant neoplasm of respiratory and digestive systems (197), Other disorders of the urethra and urinary tract (599), Iron deficiency anaemias (280), Other hypertensive heart diseases (404), Intracerebral haemorrhage (431), Paralytic ileus (560), Injury to intra-abdominal organs (866), Other diseases of the upper respiratory tract (478), Hereditary hemolytic anaemias (282), Polyarteritis nodosa and allied conditions (446)
Other Disorders of Soft Tissues (729)	eICU	Empyema (510), Aplastic anaemia (284), Poisoning by antibiotics (980), Pancreatic disorders (576), Cholelithiasis (574), Malignant neoplasm of female breast (174), Diseases of hard tissues of teeth (523), Infections of the kidney (590), Toxic effect of other substances, chiefly nonmedicinal as to source (292), Other fracture of skull (853), Malignant neoplasm of other and unspecified sites (193), Cholecystitis (575), Open wound of other and unspecified sites, except limbs (874), Other diseases of the upper respiratory tract (478), Complications affecting specified body systems (997), Fracture of vault of the skull (800), Diseases of the pancreas (577), Poisoning by analgesics, antipyretics, and antirheumatics (965) Thyroid gland disorders(242)
Malignant Neoplasm of Gallbladder and Extra-hepatic Bile Ducts (156)	eICU	Esophageal varices (456), Pancreatic disorders (576), Cholelithiasis (574), Other disorders of nervous system (349), Malignant neoplasm of female breast (174), Fracture of vertebral column without mention of spinal cord injury (805), Other diseases of lung (516), Malignant neoplasm of other and unspecified sites (161), Chronic liver disease and cirrhosis (570), Acute pericarditis (420), Chronic pulmonary heart disease (416), Inflammatory and toxic neuropathy (357), Bulbus cordis anomalies and anomalies of cardiac septal closure (746), Peritonitis (567), Other hypertensive heart disease (404), Other disorders of stomach and duodenum (537), Other disorders of adrenal glands (255), Asthma (493)
Malignant Neoplasm of Connective and Other Soft Tissue(171)	eICU	Osteomyelitis, periostitis, and other infections involving bone (730), Diverticula of the intestine (562), Pancreatic disorders (576), Malignant neoplasm of rectum, rectosigmoid junction, and anus (154), Malignant neoplasm of pancreas (157), Other vascular insufficiencies of the intestine (557), Chronic pulmonary heart disease (416), Acute pharyngitis (464), Iron deficiency anaemias (280), Chronic liver disease and cirrhosis (571), Empyema and pneumothorax (510), Paralytic ileus (560), Symptoms involving respiratory system and other chest symptoms (786), Other diseases of the endocardium (424), Acute bronchitis and bronchiolitis (466), Other diseases of the upper respiratory tract (478), Diverticulosis and diverticulitis of colon (562), Other peripheral vascular diseases (443), Other forms of chronic ischemic heart disease (411)
Aplastic Anaemia (284)	eICU	Empyema (510), Osteomyelitis, periostitis, and other infections involving bone (730), Malignant neoplasm of the bladder (188), Diseases of hard tissues of teeth (523), Other ill-defined cerebrovascular disease (437), Pyogenic arthritis (711), Other disorders of soft tissue (729), Occlusion and stenosis of precerebral arteries (433), Acute ill-defined, cerebrovascular disease (436), Other complications of procedures (998), Open wound of other and unspecified sites, except limbs (874), Other diseases of upper respiratory tract (478), Burns classified according to extent of body surface involved (946), Hereditary hemolytic anaemias (282), Diseases of the pancreas (577), Poisoning by analgesics, antipyretics, and antirheumatics (965), Polyarteritis nodosa and allied conditions (446), Thyroid gland disorders (242), Other forms of chronic ischemic heart disease (411)

continued on next page

Rare Condition (ICD-9-CM Code Level 3)	Dataset	Source Conditions (ICD-9-CM Code Level 3)
Effects of reduced temperature (991)	eICU	Empyema (510), Pneumonia, organism unspecified (481), Aplastic anaemia (284), Poisoning by antibiotics (980), Diseases of hard tissues of teeth (523), Malignant neoplasm of other and unspecified sites (161), Acute myocardial infarction (410), Other ill-defined cerebrovascular disease (437), Fracture of shaft of femur (821), Other diseases of the peripheral nervous system (358), Fracture of face bones (802), Generalised ischemic heart disease (414), Complications peculiar to certain specified procedures (996), Acute ill-defined, cerebrovascular disease (436), Complications affecting specified body systems (997), Diseases of the pancreas (577), Thyroid gland disorders (242), Acute bronchitis and bronchiolitis (466), Meningitis due to other organisms (322)
Acute Pericarditis (420)	eICU	Pneumonia, organism unspecified (481), Osteomyelitis, periostitis, and other infections involving bone (730), Esophageal varices (456), Acute myeloid leukaemia (205), Pancreatic disorders (576), Malignant neoplasm of female breast (174), Other unspecified infectious and parasitic diseases (999), Toxic effect of other substances, chiefly nonmedicinal as to source (292), Pyogenic arthritis (711), Chronic pulmonary heart disease (416), Pneumonia due to other specified bacteria (482), Other diseases of the peripheral nervous system (358), Fracture of face bones (802), Paralytic ileus (560), Injury to intra-abdominal organs (866), Other diseases of the upper respiratory tract (478), Other arterial embolism and thrombosis (444), Asthma (493), Malignant neoplasm of the stomach (151)
Acute Myeloid Leukemia (205)	eICU	Diseases of mitral and aortic valves (394), Other diseases of the central nervous system (344), Fracture of radius and ulna (813), Conduction disorders (426), Acute pericarditis (420), Chronic pulmonary heart disease (416), Fracture of femur (821), Drug-induced mental disorders (292), Pneumonia, organism unspecified (482), Injury to blood vessels of the abdomen (864), Fracture of face bones (802), Other symptoms involving head and neck (478), Hypertensive heart disease (402), Diseases of white blood cells (288), Other diseases of the blood and blood-forming organs (282), Gastric ulcer (531), Other disorders of the circulatory system (443), Thyroid gland disorders (242), Acute bronchitis and bronchiolitis (466)
Meningitis (322)	eICU	Empyema and pneumothorax (510), Pneumonia, organism unspecified (481), Other diseases of intestines and peritoneum (562), Malignant neoplasm of the bladder (188), Other inflammatory conditions of the skin (682), Malignant neoplasm of other and unspecified sites (161), Abscess of lung and mediastinum (572), Pneumonia, organism unspecified (480), Other disorders of the circulatory system (437), Other unspecified infectious and parasitic diseases (991), Cystitis (595), Pneumonia, organism unspecified (482), Appendicitis (541), Fracture of face bones (802), Other diseases of lung (512), Other diseases of stomach and duodenum (537), Other diseases of pancreas (577), Other disorders of urethra and urinary tract (599), Duodenal ulcer (533)
Abscess of Lung and Mediastinum (513)	eICU	Empyema and pneumothorax (510), Malignant neoplasm of nasal cavities, middle ear, and accessory sinuses (192), Other diseases of blood and blood-forming organs (253), Other diseases of respiratory system (519), Malignant neoplasm of other and unspecified sites (161), Fracture of radius and ulna (813), Complications peculiar to certain specified procedures (999), Coagulation defects (286), Alcoholic psychoses (291), Acute pericarditis (420), Septicemia (711), Renal failure (586), Injury to blood vessels of abdomen (866), Complications affecting specified body systems (996), Other diseases of muscle, ligament, and fascia (359), Other disorders of circulatory system (444), Other diseases of pancreas (577), Poisoning by analgesics, antipyretics, and antirheumatics (965), Disorders of thyroid gland (242)

end of the table

References

1. Johnson, A. E. *et al.* Mimic-iii, a freely accessible critical care database. *Sci. Data* **3**, 1–9 (2016).
2. Pollard, T. J. *et al.* The eicu collaborative research database, a freely available multi-center database for critical care research. *Sci. Data* **5**, 1–13 (2018).
3. Aliferis, C. & Simon, G. Overfitting, underfitting and general model overconfidence and under-performance pitfalls and best practices in machine learning and ai. *Artif. Intell. Mach. Learn. Heal. Care Med. Sci. Best Pract. Pitfalls* 477–524 (2024).
4. Alzubaidi, L. *et al.* A survey on deep learning tools dealing with data scarcity: definitions, challenges, solutions, tips, and applications. *J. Big Data* **10**, 46 (2023).
5. Phillips, C. *et al.* Time to diagnosis for a rare disease: managing medical uncertainty. a qualitative study. *Orphanet J. Rare Dis.* **19**, 297 (2024).
6. Liu, J. *et al.* Natural history and real-world data in rare diseases: applications, limitations, and future perspectives. *The J. Clin. Pharmacol.* **62**, S38–S55 (2022).
7. Banerjee, J. *et al.* Machine learning in rare disease. *Nat. Methods* **20**, 803–814 (2023).
8. Gupta, M. *et al.* An extensive data processing pipeline for mimic-iv. In *Machine Learning for Health*, 311–325 (PMLR, 2022).
9. Harutyunyan, H., Khachatrian, H., Kale, D. C., Ver Steeg, G. & Galstyan, A. Multitask learning and benchmarking with clinical time series data. *Sci. Data* **6**, 96 (2019).
10. van de Water, R. *et al.* Yet another icu benchmark: A flexible multi-center framework for clinical ml. In *The Twelfth International Conference on Learning Representations* (2024).
11. Hyland, S. L. *et al.* Early prediction of circulatory failure in the intensive care unit using machine learning. *Nat. Medicine* **26**, 364–373 (2020).
12. Moritz, S. & Bartz-Beielstein, T. imputets: Time series missing value imputation in r. *R J.* **9** (2017).
13. Niako, N., Melgarejo, J. D., Maestre, G. E. & Vatcheva, K. P. Effects of missing data imputation methods on univariate blood pressure time series data analysis and forecasting with arima and lstm. *BMC Med. Res. Methodol.* **24**, 320 (2024).
14. Hochreiter, S. & Schmidhuber, J. Long short-term memory. *Neural Comput.* **9**, 1735–1780 (1997).
15. Zhao, Z. *et al.* Leave no patient behind: Enhancing medication recommendation for rare disease patients. In *Proceedings of the 47th International ACM SIGIR Conference on Research and Development in Information Retrieval*, 533–542 (2024).
16. Yang, C., Westover, M. B. & Sun, J. Manydg: Many-domain generalization for healthcare applications. In *11th International Conference on Learning Representations* (2023).
17. Guo, C., Pleiss, G., Sun, Y. & Weinberger, K. Q. On calibration of modern neural networks. In *International conference on machine learning*, 1321–1330 (PMLR, 2017).
18. Jiang, X., Osl, M., Kim, J. & Ohno-Machado, L. Smooth isotonic regression: a new method to calibrate predictive models. *AMIA Summits on Transl. Sci. Proc.* **2011**, 16 (2011).
